# Oral *Lacticaseibacillus rhamnosus GG* Exposure During Pregnancy and Effects on Maternal Inflammatory Response—A Blinded, Pilot Randomized, Placebo‐Controlled Study

**DOI:** 10.1111/aji.70190

**Published:** 2025-12-10

**Authors:** Mahsa Nordqvist, Maria Hallingström, Alice Nerén, Maria Bullarbo, Pihla Kuusela, Malin Barman, Pontus Thulin, Verena Sengpiel, Bo Jacobsson

**Affiliations:** ^1^ Region Västra Götaland Södra Älvsborg Hospital Department of Obstetrics and Gynecology Borås Sweden; ^2^ Region Västra Götaland, Södra Älvsborg Hospital Department of Research Education and Innovation Borås Sweden; ^3^ Department of Obstetrics and Gynecology Institute of Clinical Sciences Sahlgrenska Academy University of Gothenburg Gothenburg Sweden; ^4^ Akleja Womens Health Clinic Borås Sweden; ^5^ Registered Nurse Midwife, PhD Department of Obstetrics and Gynecology Sahlgrenska University Hospital Region Västra Götaland Gothenburg Sweden; ^6^ Department of Obstetrics and Gynecology Institute of Clinical Sciences Göteborgs Universitet Göteborgs Sweden; ^7^ Region Västra Götaland Södra Älvsborg Hospital Borås Sweden; ^8^ Region Västra Götaland Department of Obstetrics and Gynecology Södra Älvsborg Hospital Borås Sweden; ^9^ Department of Life Sciences, Chalmers University of Technology Gothenburg Sweden; ^10^ Region Västra Götaland Department of Clinical Immunology and Transfusion Medicine Sahlgrenska University Hospital Gothenburg Sweden; ^11^ Region Västra Götaland Department of Obstetrics and Gynecology Sahlgrenska University Hospital Gothenburg Sweden

## Abstract

**Problem:**

This study aimed to investigate whether *Lacticaseibacillus rhamnosus GG* intake during pregnancy influences maternal cytokine levels and lymphocyte subpopulations.

**Method:**

Pregnant women were categorized into three groups: those with a history of spontaneous preterm delivery (PTD) (*n* = 40), those with a history of preeclampsia (*n* = 40), and nulliparous or parous women without a history of PTD or preeclampsia (*n* = 40). Participants were randomized to receive either *L. rhamnosus GG* or a placebo. Maternal blood samples were collected at baseline before gestational week 19 (visit 1) and around gestational weeks 25 and 35 (visits 2 and 3).

The primary outcome was the change in tumor necrosis factor‐alpha (TNF‐α) levels in monocytes after stimulation of maternal blood with *Escherichia coli* lipopolysaccharide (LPS). Secondary outcomes included lymphocyte subpopulation analysis and changes in TNF‐α, interleukin (IL)‐10, and IL‐12 levels after stimulation with *E. coli* LPS, *Lactobacillus paracasei*, or *Pseudomonas aeruginosa*.

**Results:**

In the intention‐to‐treat analysis, no significant differences in TNF‐α levels were observed. However, a sensitivity analysis excluding participants with fever or recent antibiotic use (*n* = 27) revealed a significant decrease in TNF‐α levels in the intervention group at visit 2 compared to an increase in the placebo group (mean difference −11785 cells/mL, 95% CI: −22459 to −1287, *p* = 0.03). Secondary analyses showed lower total lymphocyte, T‐cell, IL‐10, and IL‐12 levels at visit 2 and higher IL‐10 and IL‐12 levels at visit 3 in the intervention group.

**Conclusions:**

Although *L. rhamnosus GG* did not significantly affect TNF‐α levels, its influence on lymphocyte and cytokine levels warrants further investigation through larger trials.

**Abbreviations**
BMI: body mass index
CI: confidence interval
IVF: in vitro fertilization
MoBa: the Norwegian Mother, Father and Child Cohort Study
PTD: preterm delivery
TLR: toll‐like receptor
WHO: World Health Organization

## 1 Introduction

The maternal immune system plays a pivotal role in pregnancy and the delivery process [1, 2]. Deviation from normal inflammatory adaptation may lead to pregnancy‐related complications, such as preterm delivery (PTD) and preeclampsia [3, 4, 5, 6, 7, 8, 9, 10, 11, 12, 13, 14]. These are two major challenges in modern obstetrics, with potentially devastating consequences for the mother and offspring and short‐ and long‐term adverse outcomes. Despite advancements in clinical practice, reliable preventive and therapeutic strategies for preeclampsia and spontaneous preterm delivery remain underdeveloped in modern obstetrics [15, 16, 17, 18, 19]. In this regard, probiotics have emerged as a focus of research.

In three previous publications on the Norwegian Mother, Father and Child Cohort Study (MoBa), we found promising associations between intake of probiotic‐containing milk products during pregnancy and reduced risk of spontaneous PTD and preeclampsia [20, 21, 22]. Probiotics are live microorganisms that supplement an individual's ordinary diet and confer a health benefit to the host when administered at therapeutic levels [23]. The anti‐inflammatory effect of orally ingested probiotics has been shown in vivo [24, 25]. Probiotics also have the potential to colonize the vagina [26] and normalize the bacterial flora in the lower genital tract in non‐pregnant women [27]. Nevertheless, these results could not be replicated in a study on healthy pregnant women supplemented with the probiotics *Lacticaseibacillus rhamnosus GR‐1* and *L. reuteri RC‐14* for 3 months [28]. On the other hand, there is evidence that these probiotics may counteract *Escherichia coli* lipopolysaccharide (LPS)‐stimulated inflammatory outputs in human placental trophoblast cells [29, 30]. Yang et al. reported that the supernatant (bacteriocin) of *L. rhamnosus GR‐1* attenuated LPS‐induced inflammation and PTD in pregnant mice [31]. Further, Yeganegi et al. showed that *L. rhamnosus GR‐1* had an anti‐inflammatory effect on the human trophoblast cell response to LPS exposure, whereby IL‐10 was upregulated, and LPS‐stimulated TNF‐α production was suppressed [32]. Additionally, *L. rhamnosus GG* has been observed to have T‐cell‐deactivating properties, leading to increased levels of IL‐10. In contrast, other lactobacillus strains, such as *L. paracasei*, have been found to have T‐cell‐activating tendencies, leading to higher levels of IL‐12 and lower levels of IL‐10 [33].

Cytokine dysregulation has been linked to PTD and preeclampsia [34, 35, 36, 37, 38], and it could hence be hypothesized that, during pregnancy, probiotics such as *L. rhamnosus GG* could ameliorate these dysregulations and reduce the risk of these complications. In a prospective, randomized, placebo‐controlled trial, women with preterm pre‐labor rupture of membranes (PPROM) given 1 × 10^8^ *L. rhamnosus GG* and *L. gasseri*, in addition to standard antibiotics prophylaxis, had a prolonged latency period, and neonatal outcomes were indeed improved [39]. In another randomized placebo‐controlled trial evaluating pregnancy outcomes in pregnant women with cerclage, the intervention arm receiving oral probiotics (*L. acidophilus, L. plantarun, L. fermentum*, and *L. gasseri*) from pregnancy weeks 16–37 had a reduced incidence of premature rupture of membranes [40]. However, to the best of our knowledge, no randomized, placebo‐controlled study has previously investigated changes in cytokines (TNF‐α, IL‐12, and IL‐10) and lymphocyte subpopulations in the human maternal immune response to probiotic supplementation at different time points during pregnancy. The primary aim of this randomized, placebo‐controlled pilot study was to determine whether oral intake of *L. rhamnosus GG* affects TNF‐α levels in maternal blood monocytes in response to stimulation with *E. coli* LPS at different time points during pregnancy. The secondary aim was to investigate whether *L. rhamnosus GG* affects lymphocyte subpopulations and IL‐10 and IL‐12 levels, as well as to analyze whether a history of spontaneous PTD or preeclampsia modifies the impact on the inflammatory response.

## 2 Methods

### 2.1 Population and Study Design

Pregnant women aged 18 or older with singleton pregnancy before gestational week 19 according to menstrual data or sonography were eligible for inclusion. The participants were recruited upon their first visit to maternal care units in western Sweden and the Departments of Obstetrics and Gynecology, Sahlgrenska University Hospital, Gothenburg, between June 2012 and December 2019, mainly during the first trimester. The exclusion criteria were age younger than 18, having a chronic disease or uterine malformation, taking immunomodulatory medication or hormonal treatment, unwillingness to discontinue other products containing probiotics during study time, and unable to provide written informed consent.

Three subgroups of pregnant women were included based on the findings of Amory et al. [41], indicating that women with previous spontaneous PTD have higher inflammatory responses to LPS stimulation than women with previous normal pregnancies.

The three subgroups were: (a) spontaneous PTD (at < 37 gestational weeks), starting with preterm labor or PPROM, in a previous pregnancy (*n* = 40); (b) preeclampsia in a previous pregnancy, regardless of term or iatrogenic preterm labor due to preeclampsia (*n* = 40); and c) nulliparous women or parous women with no history of preeclampsia or spontaneous PTD, referred to as the control subgroup (*n* = 40). We chose to include a subgroup with a history of preeclampsia since preeclampsia is considered an inflammatory disease [42], and our previous findings indicate an association between probiotic intake and decreased risk of preeclampsia and PTD [28, 29, 30]. At the time of this study, preeclampsia was defined as hypertension (blood pressure ≥140/90) and proteinuria (≥ 300 mg/24 h), with onset after 20 weeks of gestation [43].

Participants were asked to refrain from taking other products containing probiotics during pregnancy. The initial recruitment visit (visit 1) was denoted as “baseline,” including pregnancies before gestational week 19. After randomization, the women were asked to take the probiotic intervention or placebo capsules from baseline until labor. Subsequent visits were scheduled around gestational weeks 25 and 35 (visits 2 and 3, respectively) with a research midwife at the Departments of Obstetrics and Gynecology, Sahlgrenska University Hospital. Blood samples were taken at each of the three visits. Maternal characteristics and pregnancy outcomes were obtained from the patient charts.

### 2.2 Randomization

The women were randomly assigned to receive either a probiotic intervention or a placebo in a double‐blinded fashion. Randomization was performed by a computerized randomization tool. Each participant was assigned a sequential number and received capsules in boxes marked with the corresponding number. All the capsules were supplied by Fuko Pharma OY/AB, Finland, who also provided a code key containing the serial numbers and their respective codes (either A or B), along with a separate document defining A and B as placebo or intervention. The code key remained undisclosed to a representative of the University of Gothenburg until the statistical analysis plan was finalized. The definitions of A and B were not revealed until all analyses were completed. The group that received the active ingredient, i.e., probiotic, is hereafter referred to as the intervention arm.

### 2.3 Intervention

The commercial name of the probiotic intervention is Biophilus Strong, which is only marketed in Finland and is registered as a food supplement. These capsules contain maltodextrin from corn, *L. rhamnosus GG*, and magnesium stearate (anti‐caking agent). The placebo capsules were visually identical and had the same content, with the exception of *L. rhamnosus GG*, which was replaced by maltodextrin to make the weight equal. Women were required to take one capsule twice a day until delivery, yielding a daily *L. rhamnosus GG* intake of at least one billion bacteria. The manufacturer continuously ensured the viability of the bacteria. The capsules were stored at room temperature, according to the manufacturer's instructions. Participant compliance was assessed with questionnaires administered at each visit. The remaining capsules that had not been consumed by the time of delivery were also collected. As iron intake may interfere with probiotic uptake, women taking a form of iron supplement a

### 2.4 Sampling

Venous blood samples were collected at each of the three visits in EDTA tubes and heparin tubes and immediately transported at room temperature. All analyses were performed at the Department of Clinical Immunology, Sahlgrenska University Hospital, Gothenburg, Sweden. Analysis of the maternal blood samples is described in Supporting Information, File 1, and the gating strategy in the case of flow cytometry is shown in Supporting Information, File 2.

### 2.5 Statistics

#### 2.5.1 Primary Analysis

**FIGURE 1:**
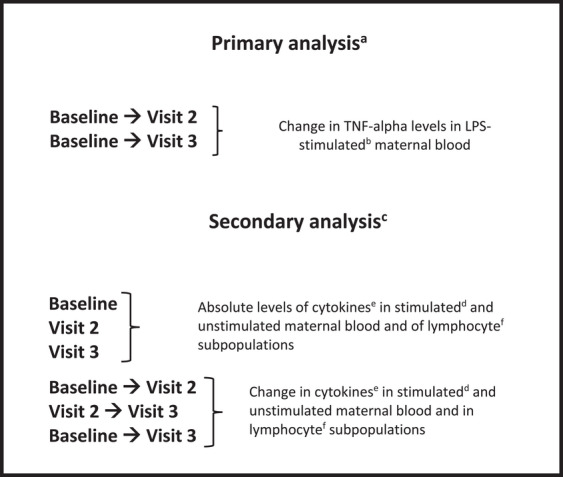
Overview of analyses and main results. (a) Performed on intention‐to‐treat population (*n* = 105) by randomization. (b) Stimulated with *Escherichia coli* lipopolysaccharide (LPS). c) Performed on intention‐to‐treat population (*n* = 105) and within each subgroup by randomization. (d) Stimulated with lipopolysaccharide (LPS) from *Escherichia coli*, or with *Lactobacillus paracasei* or *Pseudomonas aeruginosa*. (e) TNF‐alpha, IL‐10, IL‐12 (number of cytokine‐positive monocytes/mL). (f) Total lymphocytes, T‐cells, T‐cell%, helper T‐cells, helper T‐cell%, cytotoxic T‐cells, cytotoxic T‐cell%, NK cells, NK cell%, B‐cells, B‐cell%, helper T‐cells/cytotoxic T‐cells, regulatory T‐cell% of helper T‐cells, regulatory T‐cell% of lymphocytes. The baseline visit occurred around gestational week 8, and visits 2 and 3 occurred around gestational weeks 25 and 35, respectively.

The outcome measures in the primary analysis were the respective changes in TNF‐α levels from baseline to visit 2 and from baseline to visit 3 in maternal blood stimulated with *E. coli* LPS (reported as the number of cytokine‐positive monocytes/mL). The primary analysis was performed on the intention‐to‐treat (ITT) population (*n* = 105; including all three subgroups: previous PTD, previous preeclampsia, and control) by randomization (Figure 1).

#### 2.5.2 Secondary Analyses

Given that this study is primarily exploratory in nature, secondary analyses were conducted independently of the outcomes of the primary analysis (Figure 1). The secondary analysis outcome measures were lymphocyte subpopulations measured at all visits and changes in values between the three visits. Outcomes were presented for the ITT population (*n* = 105) and each of the three subgroups, by randomized group, as well as absolute values of TNF‐α, IL‐10, and IL‐12 in unstimulated and stimulated maternal blood, stimulated with *E. coli* LPS, *Lactobacillus paracasei*, or *Pseudomonas aeruginosa*. Analyzed lymphocyte subpopulations comprise total lymphocytes, T‐cells, T‐cell %, helper T‐cells, helper T‐cell %, cytotoxic T‐cells, cytotoxic T‐cell %, NK cells, NK cell %, B‐cells, B‐cell %, helper T‐cells/cytotoxic T‐cells, regulatory T‐cell % of helper T‐cells, and regulatory T‐cell % of lymphocytes.

The primary analysis compared the intervention and placebo arms at a significance level of alpha 0.05/2 = 0.025, according to the Bonferroni adjustment. All other analyses were considered exploratory, and *p* values were provided for descriptive purposes.

As acute infection could be a mediator of the probiotic effect on pregnancy outcome, we included all participants in the main analyses but decided a priori to perform sensitivity analyses excluding participants with acute infections, as the expected changes in the maternal inflammatory response due to gestational age might be much smaller than changes in the inflammatory response due to acute infection.

#### 2.5.3 Definition of Analysis Populations

According to our statistical analysis plan and “clean file” meeting, results are presented for the ITT population, defined as all women who provided a blood sample at baseline and visit 2, since not all participants included have outcome data (cytokine and lymphocyte subpopulation levels). A per‐protocol analysis, comparing baseline variables and the primary analysis, was performed, excluding women with <90% compliance.

### 2.6 Statistical Methods

Continuous variables were presented with mean, standard deviation (SD), median, Q1, Q3, minimum, and maximum, while categorical variables were given with number and percentage. In the case of dichotomous and continuous outcome variables, differences between groups/arms were presented with mean differences, 95% confidence intervals (CI), and *p* values (within‐group/arm and between groups/arms).

Fisher's exact test was used for dichotomous variables, and Fisher's non‐parametric permutation test for continuous variables. Pearson's chi‐squared test was used for nominally scaled variables. Fisher's non‐parametric permutation test for paired observations was used for changes within and between groups/arms. Baseline variables that differed between the randomized arms and affected the outcome variables were considered confounders in adjusted multivariable analyses using analysis of covariance. Since the only significant difference in maternal characteristics was body mass index (BMI) in the spontaneous PTD and control subgroups, adjustment for BMI was only performed in the three subgroups and not in the analysis of the ITT population. In the unadjusted subgroup analysis, Student's *t*‐test was used for comparing independent groups/arms for continuous variables, as well as for paired observations within a group/arm. In the statistical analysis of cytokine and subpopulation of lymphocyte variables, the changes in the variables were checked for normality and were not found to be strongly positively skewed. However, both means and medians were presented to illustrate the actual spread and effect of possible outliers. Outliers were not excluded in order to illustrate the actual natural spread of cytokines. Cytokine levels of TNF‐α, IL‐10, and IL‐12 were analyzed in monocytes in maternal blood before and after stimulation with *E. coli* LPS or strains of *L. paracasei* or *P. aeruginosa*. Cytokine levels in stimulated maternal blood were compared to levels before stimulation (hereafter, unstimulated) by calculating differences and ratios. Respective changes in cytokine levels between the three visits were calculated. For further details, please refer to the supplemental methods section.

Following enrollment on ClinicalTrials.gov (NCT02693041), in the “clean file” meeting, when signing the analysis plan and before the database was locked, the decision was taken to partition the principal outcome into both primary and secondary outcomes, as delineated subsequently. As no previous data were available, the present study was not preceded by a power calculation and was performed as an experimental pilot randomized controlled trial.

Data that are not presented in the paper can be provided upon request.

## 3 Results

A summary of the results is presented in Table S1, and an illustrative overview of cytokine responses is shown in Figures S1 and S2.

### 3.1 Study population

**FIGURE 2:**
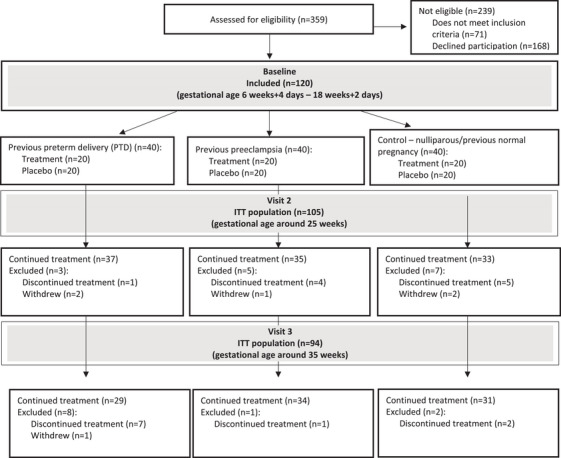
Participant flow chart. Reasons for discontinuing treatment were hyperemesis (*n* = 3), miscarriage (*n* = 4), undiagnosed twin pregnancy at baseline (*n* = 2), preterm delivery (*n* = 9), preeclampsia (*n* = 1), and side effects (*n* = 1). ITT, intention‐to‐treat.

A total of 359 women were assessed for eligibility (Figure 2). Of these, 288 women fulfilled the inclusion criteria, while 168 women declined to participate. Consequently, 120 women were enrolled in the study, with an equal distribution of 40 women in each of the three subgroups (previous PTD, previous preeclampsia, and control). The gestational age at inclusion ranged from 6 weeks + 4 days to 18 weeks + 2 days. Fifteen women were excluded after the baseline assessment, leaving 105 women in the ITT population. An additional 11 women were excluded during visit 3. Reasons for discontinuing treatment are presented in Figure 2. Ultimately, 94 participants completed all three visits (29 women in the previous PTD group, 34 in the previous preeclampsia group, and 31 in the control group).

### 3.2 Maternal Characteristics and Delivery Outcomes

In the ITT population, maternal characteristics and delivery outcomes did not differ between the intervention and placebo arms (Table 1). However, in the previous PTD subgroup, the placebo arm had a significantly higher BMI (mean difference in BMI −3.48, 95% CI: −6.40 to −0.57, *p* = 0.02). In the control subgroup, the intervention arm had a significantly higher BMI (mean difference in BMI 2.79, 95% CI: 0.19–5.51, *p* = 0.037).

**TABLE 1:** Maternal characteristics at baseline and delivery outcomes (ITT^a^, *n* = 105).

Variable	Total (*n* = 105)	Intervention (*n* = 53)	Placebo (*n* = 52)	*p* value
**Maternal age (years)**	31.9 (4.8)	32.3 (5.0)	31.5 (4.6)	0.41
	32 (22; 43)	32 (23; 43)	32 (22; 40)	
	(28; 35)	(29; 36)	(28; 35)	
	*n* = 104	*n* = 53	*n* = 51	
**Maternal body mass index**	24.6 (4.6)	24.6 (4.5)	24.6 (4.8)	0.95
	23.4 (17.4; 40.6)	23.3 (18; 36.9)	23.6 (17.4; 40.6)	
	(21.4; 26.8)	(20.9; 27.7)	(21.8; 25.8)	
	*n* = 104	*n* = 52	*n* = 52	
**Ethnicity**				
Caucasian	96 (93.2%)	48 (92.3%)	48 (94.1%)	
**Marital status, partner**	104 (99.0%)	52 (98.1%)	52 (100.0%)	
**Parous, parity**	94 (89.5%)	46 (86.8%)	48 (92.3%)	0.55
**Prior preterm delivery**	48 (45.7%)	25 (47.2%)	23 (44.2%)	0.92
**Prior spontaneous preterm delivery**	39 (37.5%)	19 (36.5%)	20 (38.5%)	1.00
**Prior spontaneous extremely early delivery, < 28 gestational weeks**	<3 (<3%)	<3 (<3%)	0 (0.0%)	1.00
**Previous late spontaneous abortion, >13 gestational weeks**	3 (3.0%)	<3 (<3%)	2 (4.0%)	0.98
**Smoking before and during pregnancy**	7 (6.7%)	4 (7.7%)	3 (5.8%)	1.00
**In vitro fertilization**	8 (8.8%)	3 (6.4%)	5 (11.4%)	0.64
**Chronic disease at baseline**	21 (20.0%)	9 (17.0%)	12 (23.1%)	0.59
**Hypertensive disease at baseline**	2 (1.9%)	0 (0.0%)	2 (3.8%)	0.49
**Severe preeclampsia in previous pregnancy**	16 (18.8%)	7 (18.4%)	9 (19.1%)	1.00
**Acetylsalicylic acid prophylaxis current pregnancy**	10 (9.5%)	6 (11.3%)	4 (7.7%)	0.77
**Birth weight (gram)**		3419 (592)	3511 (578)	0.42
		3473 (1585; 4480)	3484 (2020; 4708)	
		(3115; 3758)	(3230; 3933)	
		*n* = 52	*n* = 52	
**1‐min Apgar <7 points**		<3 (<3%)	<3 (<3%)	1.00
**Placental weight (gram)**		616 (102)	600 (118)	0.51
		617 (410; 860)	591 (365; 855)	
		(532; 670)	(510; 680)	
		*n* = 44	*n* = 38	
**Neonatal intensive care unit admission**		9 (17.6%)	7 (14.9%)	0.93
**Indicated preterm delivery current pregnancy**		0 (0.0%)	<3 (<3%)	0.99
**Spontaneous preterm delivery current pregnancy**		7 (13.2%)	3 (5.8%)	0.33
**Gestational hypertension current pregnancy**		3 (5.7%)	2 (3.9%)	1.00
**Preeclampsia current pregnancy**		5 (9.4%)	3 (5.8%)	0.74
**Gestational age at delivery, days**		272.8 (15.9) 275 (209; 296) (269; 282) *n* = 53	274.0 (13.5) 276 (217; 294) (268.5; 279.5) *n* = 52	0.67
**Infant's sex, female**		26 (53.1%)	28 (54.9%)	1.00

*Note:* For categorical variables, *n* (%) is presented. For continuous variables, mean, median (min; max) / (Q1; Q3) / *n* are presented. For comparison between groups, Fisher's exact test (lowest 1‐sided *p* value multiplied by 2) was used for dichotomous variables; chi‐squared test was used for non‐ordered categorical variables; and Fisher's non‐parametric permutation test was used for continuous variables. The confidence intervals for dichotomous variables correspond to the unconditional exact confidence limits. If no exact limits could be computed, the asymptotic Wald confidence limits with continuity correction were calculated instead. The confidence intervals for the mean differences between groups are based on Fisher's non‐parametric permutation test. Variables where data were available for fewer than 3 individuals are reported as “<3 (<3%)” in order to protect privacy.

^a^ Intention‐to‐treat.

At the three study visits, there were no differences in blood pressure between the intervention and placebo arms, either in the ITT population (*n* = 105) or in the three subgroups (data not presented). No significant differences were found regarding gestational age at each visit, on analysis of occasional intake of other probiotic products, iron supplementation, or antibiotics intake and/or fever one week before each visit, either in the ITT population (Table S2) or in the three subgroups (data not presented). Individuals who consumed other probiotic products were classified as minor violators owing to the infrequent nature of their reported consumption.

### 3.3 Primary Analysis: TNF‐α Outcomes With and Without LPS Stimulation

**FIGURE 3:**
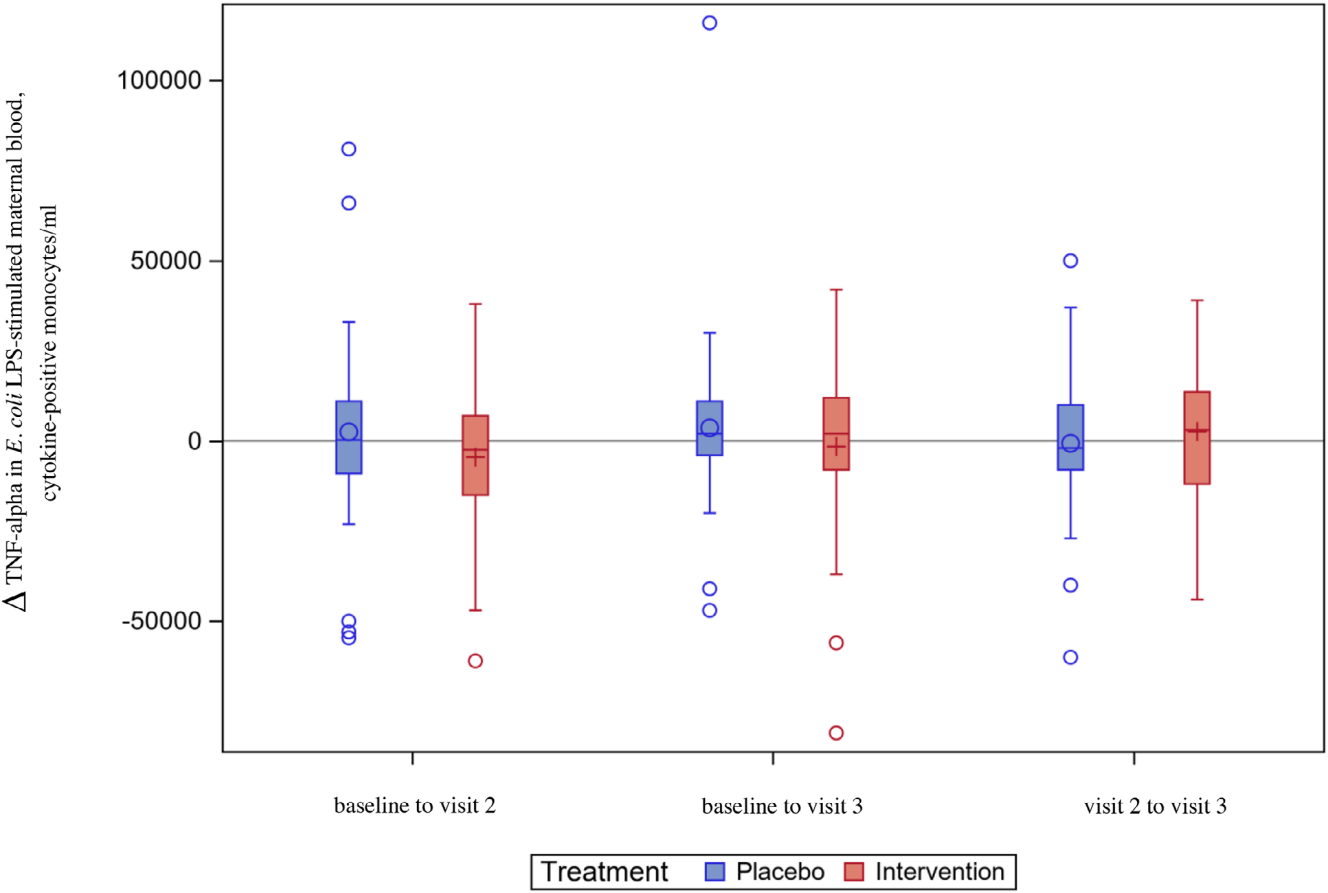
TNF‐α^†^ levels in E. coli^§^ LPS^‡^‐stimulated maternal blood – changes over time (ITT*^¶^*, *n* = 105). Boxplot diagram of the changes in TNF‐a levels in maternal blood stimulated with *E. coli* LPS from baseline to visit 2, baseline to visit 3, and visit 2 to visit 3, showing no significant difference between the treatment arms. Analyses were performed in the ITT population. The box represents Q1 to Q3. The vertical line in the box is the median. The circle (in the placebo arm) or the plus sign (in the intervention arm) represents the mean. The whiskers represent the 1.5 interquartile range from Q1 and Q3; values above or below are shown as individual circles. † Tumor necrosis factor‐alpha. ‡ Lipopolysaccharide. § *Escherichia coli*. ^¶^ Intention‐to‐treat.

In the ITT population, there were no statistically significant differences between the intervention and placebo arms in the analysis of TNF‐α levels in maternal blood with and without stimulation by *E. coli* LPS at any visit or based on changes in TNF‐α levels between visits (Figure 3 and Table S3).

Regarding secondary outcomes, there were no significant differences in the analysis of TNF‐α levels in maternal blood with and without stimulation by *L. paracasei, E. coli* LPS, or *P. aeruginosa*, either in the ITT population (*n* = 105) or in the three subgroups (data not presented).

### 3.4 Results From Secondary Analysis

#### 3.4.1 Lymphocyte Subpopulations

##### 3.4.1.1 Intention‐to‐Treat Population (ITT, *n* = 105)

The percentage of T‐cells (T‐cell %) increased in the intervention and placebo arms from baseline to visits 2 and 3. The elevation was greater in the intervention arm (Table S4a). No other significant differences were found regarding lymphocyte subpopulations (data not presented).

##### 3.4.1.2 Previous PTD, Previous Preeclampsia, and Control Subgroups

In the previous PTD subgroup, no significant differences were found in lymphocyte populations between the intervention and placebo arms (data not presented). In the previous preeclampsia subgroup, the intervention arm had lower lymphocyte levels at visit 2. Moreover, T‐cell levels were lower in the intervention arm at visit 2. When subgroups of T‐cells were analyzed separately, lower levels of helper T‐cells were found in the intervention arm at all three visits (Table S4b). In the control subgroup, a decrease in the percentage of regulatory T‐cells was observed at visit 3 in the intervention arm compared to the placebo arm. Furthermore, the percentage of regulatory T‐cells decreased from visit 2 to visit 3 in the intervention arm, contrasting with an increase in the placebo arm (Table S4c). No other significant differences were found regarding lymphocyte subpopulations (data not presented).

#### 3.4.2 IL‐10

##### 3.4.2.1 Intention‐to‐Treat Population (ITT, *n* = 105)

**FIGURE 4:**
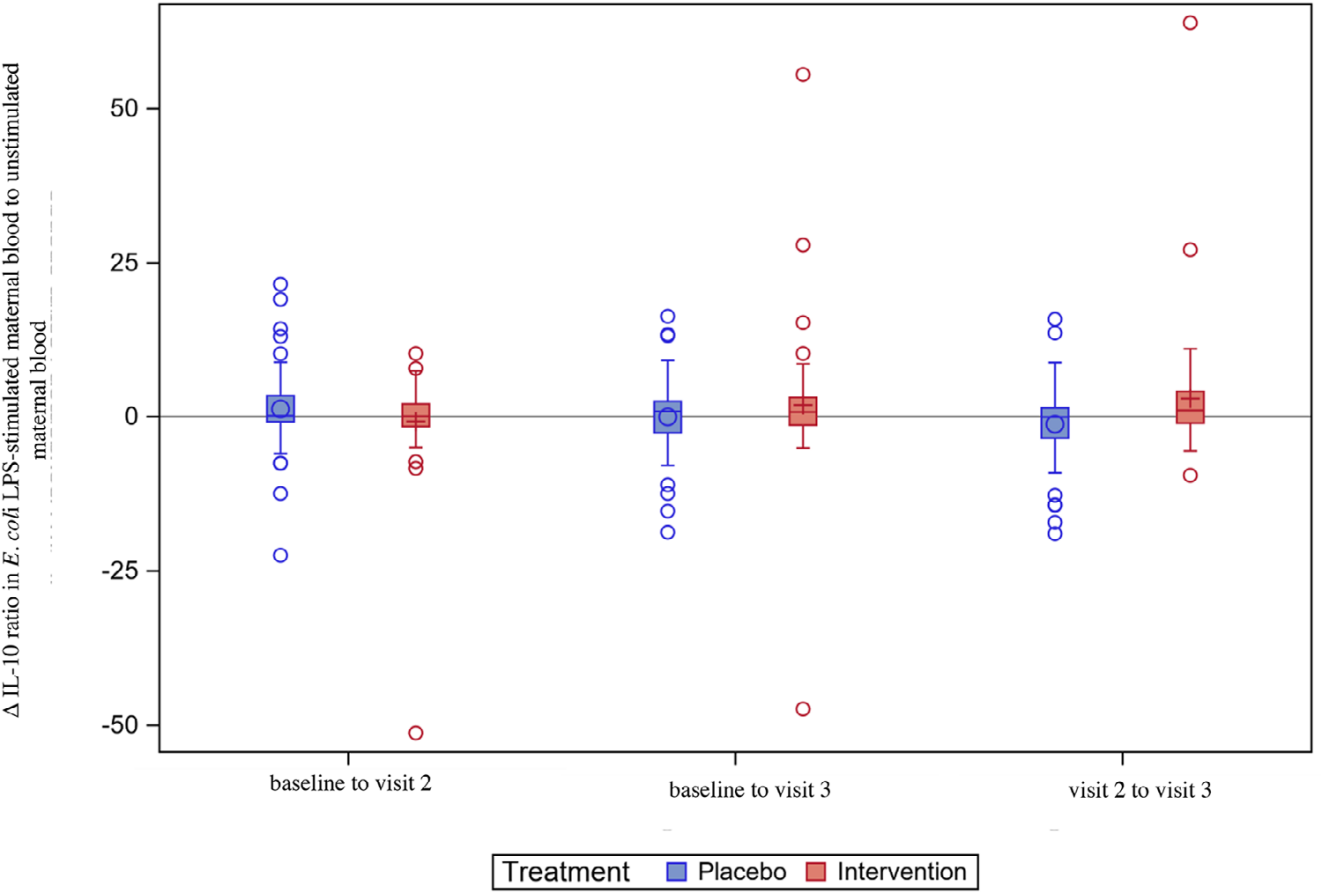
Ratio of IL‐10^†^ levels in *Pseudomonas aeruginosa—*stimulated maternal blood and unstimulated maternal blood*—*changes over time (ITT‡, *n* = 105). Boxplot diagram of the ratio of IL‐10 levels in *E. coli* LPS‐stimulated maternal blood and unstimulated maternal blood, showing an increasing ratio in the intervention arm and a decreasing ratio in the placebo arm from visit 2 to visit 3, *p* = 0.024. Analyses were performed in the ITT population. The box represents Q1 to Q3. The vertical line in the box is the median. The circle (in the placebo arm) or the plus sign (in the intervention arm) represents the mean. The whiskers represent the 1.5 interquartile range from Q1 and Q3; values above or below are shown as individual circles. † Interleukin‐10. ‡ Intention‐to‐treat.

In the intervention arm, the mean and median ratios of IL‐10 in *P. aeruginosa*‐stimulated and unstimulated maternal blood increased from visit 2 to visit 3. In contrast, these ratios decreased in the placebo arm. The mean difference between the two arms was 4.14 adjusted mean cells/mL (95% CI: 0.45–7.98, *p* = 0.024) (Figure 4 and Table S5a). There were no other significant differences between the intervention and placebo arms (data not presented).

##### 3.4.2.2 Previous PTD, Previous Preeclampsia, and Control Subgroups

In the previous PTD subgroup, the mean and median levels of IL‐10 in unstimulated maternal blood decreased from visit 2 to visit 3 in the intervention arm, while they increased in the placebo arm. The difference between IL‐10 levels in *P. aeruginosa*‐stimulated and unstimulated maternal blood decreased from baseline to visit 2 in the intervention arm and increased in the placebo arm. Moreover, the ratio between the mean and median levels of IL‐10 in *P. aeruginosa*‐stimulated and unstimulated maternal blood at baseline and visit 2 decreased in the intervention arm and increased in the placebo arm (Table S5b).

There were no other significant differences between the intervention and placebo arms (data not presented).

#### 3.4.3 IL‐12

##### 3.4.3.1 Intention‐to‐treat population (ITT, *n* = 105)

**FIGURE 5:**
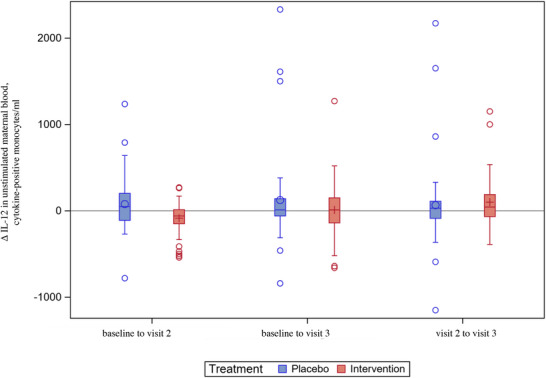
IL‐12^†^ levels in unstimulated maternal blood – changes over time (ITT^‡^, *n* = 105). Boxplot diagram of IL‐12 levels in unstimulated maternal blood, showing decreasing levels from baseline to visit 2 in the intervention arm and increasing levels in the placebo arm, *p* = 0.0009. Analyses were performed in the ITT population. The box represents Q1 to Q3. The vertical line in the box is the median. The circle (in the placebo arm) or the plus sign (in the intervention arm) represents the mean. The whiskers represent the 1.5 interquartile range from Q1 and Q3; values above or below are shown as individual circles. † Interleukin‐12. ‡ Intention‐to‐treat.

**FIGURE 6:**
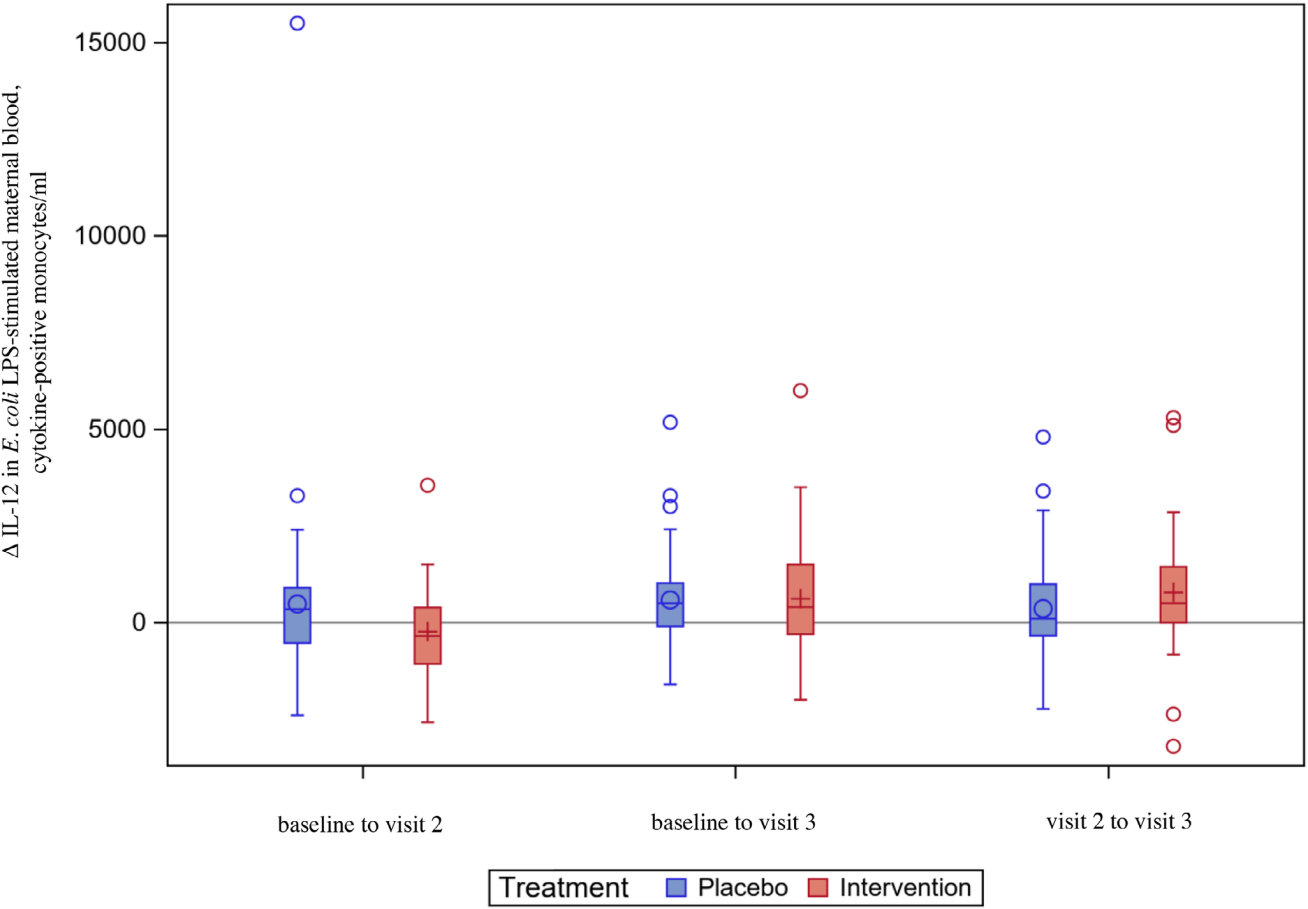
IL‐12^†^ levels in E. coli^§^ LPS^‡^‐stimulated maternal blood – changes over time (ITT^¶^, *n* = 105). Boxplot diagram of IL‐12 levels in *E. coli* LPS‐stimulated maternal blood, showing decreasing levels from baseline to visit 2 in the intervention arm and increasing levels in the placebo arm, *p* = 0.038. Analyses were performed in the ITT population. The box represents Q1 to Q3. The vertical line in the box is the median. The circle (in the placebo arm) or the plus sign (in the intervention arm) represents the mean. The whiskers represent the 1.5 interquartile range from Q1 and Q3; values above or below are shown as individual circles. † Interleukin‐12. ‡ Lipopolysaccharide. § *Escherichia coli*. ¶ Intention‐to‐treat.

The levels of IL‐12 in unstimulated maternal blood were lower at visit 2 in the intervention arm as compared to the placebo arm (Figure S3). Furthermore, the levels of IL‐12 in unstimulated maternal blood decreased from baseline to visit 2 in the intervention arm, whereas they increased in the placebo arm (Figure 5 and Table S6a). In the intervention arm, the absolute levels of IL‐12 in LPS‐stimulated maternal blood decreased from baseline to visit 2, whereas they increased in the placebo arm (Figure 6 and Table S6a).

Following stimulation of maternal blood with *L. paracasei*, IL‐12 levels in the intervention arm decreased from baseline to visit 2, while they increased in the placebo arm (Figure S4 and Table S6a). Likewise, the levels of IL‐12 in *L. paracasei*‐stimulated maternal blood decreased from baseline to visit 3 in the intervention arm, whereas they increased in the placebo arm (Table S6a).

The intervention arm had significantly lower levels of IL‐12 in *P. aeruginosa*‐stimulated maternal blood at visit 2 (adjusted mean cells/mL difference −1131, 95% CI: −2066 to −210, *p* = 0.014) (Figure S5 and Table S6a). The change in absolute levels of IL‐12 in *P. aeruginosa‐*stimulated maternal blood from visit 2 to visit 3 was significantly higher in the intervention arm than in the placebo arm (adjusted mean cells/mL difference 1053, 95% CI: 27–2077, *p* = 0.044) (Figure S6 and Table S6a).

**FIGURE 7:**
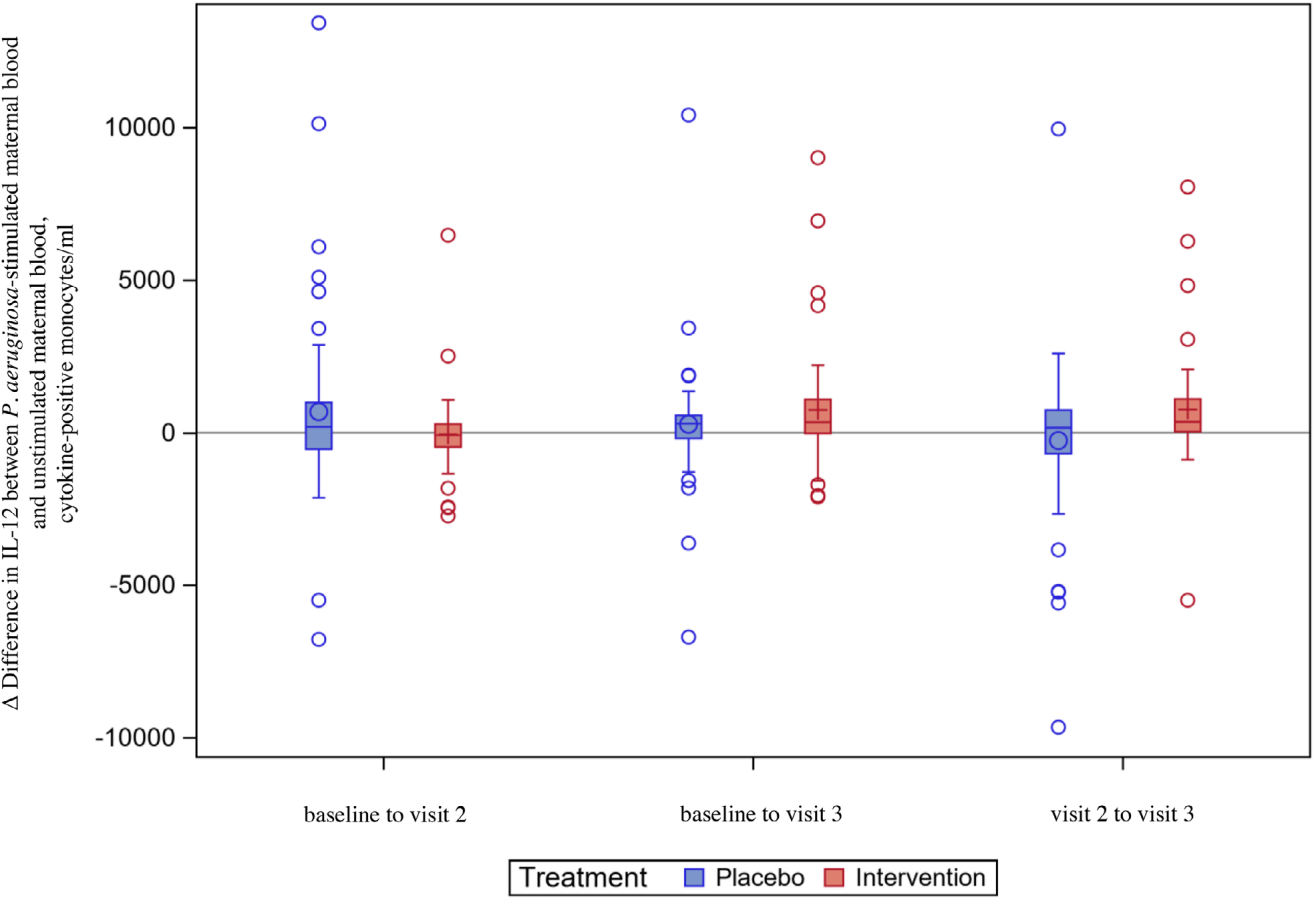
Difference in IL‐12^†^ levels in *P. aeruginosa* ^§^‐stimulated maternal blood and unstimulated maternal blood – changes over time (ITT^‡^, *n* = 105). Boxplot diagram illustrating the difference in IL‐12 levels in *P. aeruginosa*‐stimulated maternal blood and unstimulated maternal blood, showing a significantly higher difference from visit 2 to visit 3 in the intervention arm compared to the placebo arm, *p* = 0.043. Analyses were performed in the ITT population. The box represents Q1 to Q3. The vertical line in the box is the median. The circle (in the placebo arm) or the plus sign (in the intervention arm) represents the mean. The whiskers represent the 1.5 interquartile range from Q1 and Q3; values above or below are shown as individual circles. † Interleukin‐12. § Pseudomonas aeruginosa. ‡ Intention‐to‐treat.

At visit 2, the difference between IL‐12 levels in *P. aeruginosa*‐stimulated and unstimulated maternal blood was greater in the placebo arm than in the intervention arm. From visit 2 to visit 3, the difference between IL‐12 levels in *P. aeruginosa*‐stimulated and unstimulated maternal blood decreased in the placebo arm and increased in the intervention arm (adjusted mean cells/mL difference between the arms: 1011, 95% CI: 32–1986, *p* = 0.043) (Figure 7 and Table S6a).

There were no other significant differences between the intervention and placebo arms (data not presented).

##### 3.4.3.2 Previous PTD, Previous Preeclampsia, and Control Subgroups

In the previous PTD subgroup, the intervention arm exhibited lower levels of IL‐12 in unstimulated maternal blood at visit 2, as compared to the placebo arm (Table S5b). In this subgroup, the absolute levels of IL‐12 in *L. paracasei*‐stimulated maternal blood decreased from baseline to visit 2 in the intervention arm and increased in the placebo arm (adjusted mean cells/mL difference between arms: −751, 95% CI: −1477 to −25, *p* = 0.043) (Table S6b).

In the previous preeclampsia subgroup, no significant changes were found in IL‐12 levels (data not presented). In the control subgroup, the median and mean change in the ratio of IL‐12 levels in unstimulated and *P. aeruginosa*‐stimulated maternal blood from visit 2 to visit 3 increased in the intervention arm and decreased in the placebo arm (adjusted mean cells/mL difference 15.9, 95% CI: 1.9–29.9, *p* = 0.027) (Table S6c).

No other significant differences were found between the intervention and placebo arms (data not presented).

#### 3.4.4 Per‐Protocol Analysis

In the per‐protocol analysis, which excluded women with a compliance rate of < 90%, baseline variables and primary analysis results were similar to those of the ITT analyses. Three women in the intervention arm and two in the placebo arm were excluded (data not presented).

#### 3.4.5 Sensitivity Analysis

A sensitivity analysis was performed, excluding participants with fever and/or antibiotic treatment within 1 week before any of the three visits (*n* = 27). The mean and median levels of TNF‐α in *E.coli* LPS‐stimulated maternal blood decreased from baseline to visit 2 in the intervention arm and increased in the placebo arm (mean cells/mL difference −11785, 95% CI: −22459 to −1287, *p* = 0.027). The mean and median differences in TNF‐α levels in *E. coli* LPS‐stimulated and unstimulated maternal blood decreased from baseline to visit 2 in the intervention arm and increased in the placebo arm (adjusted mean cells/mL, −11765, 95% CI: −22377 to −1304, *p* = 0.027) (data not presented).

## 4 Discussion

To the best of our knowledge, this is the first pilot randomized placebo‐controlled study investigating the impact of oral *L. rhamnosus GG* supplementation on the maternal inflammatory response at different time points during pregnancy. No significant differences were found between the *L. rhamnosus GG* intervention arm and the placebo arm in our primary analysis of TNF‐α levels after *E. coli* LPS stimulation of maternal blood. However, in the sensitivity analysis excluding participants with fever and/or antibiotic treatment within one week before any of the three visits, lower TNF‐α levels were found in *E. coli* LPS‐stimulated maternal blood in mid‐pregnancy in the intervention arm. In the exploratory analyses, we found lower levels of total lymphocytes and T‐cells and generally lower IL‐10 and IL‐12 levels in the intervention arm in mid‐pregnancy and higher IL‐10 and IL‐12 levels in late pregnancy compared to the placebo arm. These results suggest that *L. rhamnosus GG* might modulate the inflammatory response in mid‐ and late pregnancy, encouraging further research.

Under healthy conditions, a pregnancy starts with an inflammatory phase during implantation [1, 2]. The next phase is predominantly anti‐inflammatory, with higher immune tolerance to allow rapid growth of the fetus [1, 2]. In the third and final phase, a pro‐inflammatory immune response plays a role in cervical ripening, promoting the contraction of the uterus and the rejection of the placenta after birth [1, 2]. In line with the immunological adaptations that occur throughout pregnancy, the proportion of T cells increased with advancing gestational age. This likely reflects the gradual modulation of maternal immune tolerance required to sustain pregnancy. The greater increase observed in the intervention group may indicate an enhanced or accelerated restoration of immune balance compared to controls. Lower levels of pro‐inflammatory IL‐12 were found in the intervention arm in mid‐pregnancy and higher levels in late pregnancy compared to the placebo arm. Consistent changes, aligning with those in healthy pregnancies, were seen for the anti‐inflammatory cytokine IL‐10. Previous studies have shown that maternal serum IL‐10 concentrations significantly decreased between the first and second trimesters [44], whereas concentrations were significantly higher in the third, compared to the first [45] and second [46] trimesters. In vitro studies demonstrate that *L. rhamnosus GG* stimulation enhances intracellular monocyte production of TNF‐α, IL‐12, and IL‐10. [47, 48] However, our findings indicate that probiotic intake during pregnancy does not consistently affect cytokine levels, exhibiting variable effects across gestational timepoints, potentially reflecting the natural cytokine fluctuations of a normal pregnancy.

While this pilot randomized controlled study did not aim to evaluate clinical outcomes, we were motivated to investigate various subgroups of pregnant women. Amory et al. [41] have demonstrated that previous spontaneous PTD, compared to previous normal delivery, may indicate an altered response to bacterial stimulation. Additionally, our research group has published three epidemiological observational cohort studies, showing that the consumption of probiotics during pregnancy was associated with a reduced risk of spontaneous PTD and preeclampsia and that the timing of probiotic consumption might play a role [20, 21, 22].

PTD is clinically divided into spontaneous PTD, including preterm labor and PPROM, and indicated PTD. Increased inflammatory response plays a role in spontaneous PTD [9, 10, 12, 14]. It has been shown that elevated IL‐12 levels in mid‐pregnancy are associated with PTD with chorioamnionitis before 35 weeks of gestation [49, 50]. In this study, mid‐pregnancy analyses (ITT and prior PTD subgroup) showed lower IL‐12 levels in the intervention arm versus placebo, both in unstimulated maternal blood and after stimulation with *E. coli* LPS, *L. paracasei*, or *P. aeruginosa*. In past epidemiological studies, our group found an association between probiotic intake and decreased risk of PTD, especially concerning intake during the first half of pregnancy [21, 22]. Probiotic suppression of mid‐pregnancy IL‐12 levels could hence be a possible pathway to a lower risk of spontaneous PTD.

In the ITT (*n* = 105), when maternal blood was stimulated with *P. aeruginosa*, mimicking infection during pregnancy, IL‐10 levels were found to increase from mid‐pregnancy to late pregnancy in the intervention arm. IL‐10 is an anti‐inflammatory cytokine. In placental tissue, reduced levels of the anti‐inflammatory cytokine IL‐10 are associated with both chorioamnionitis‐induced preterm labor and term labor [34]. However, in the previous PTD subgroup, the levels of IL‐10 in unstimulated maternal blood, mimicking a regular pregnancy, decreased from mid‐pregnancy to late pregnancy in the intervention arm. It has been shown that PTD without intra‐amniotic infection is associated with higher IL‐10 levels in the amniotic fluid than in term delivery [35]. Furthermore, IL‐10 expression is higher in the cervical squamous epithelium in cases of preterm labor than at term labor [36]. It could thus be hypothesized that probiotic suppression of IL‐10 levels during late pregnancy, in the absence of infection, might lower the risk of PTD.

The maternal inflammatory response is crucial in the development of preeclampsia. Insufficient spiral arterial remodeling is thought to be the initial phase of this condition [51, 52], leading to placental ischemia and inflammation, causing endothelial dysfunction [51, 52]. It has been shown that placental hypoxia is associated with decreased levels of IL‐10, concurrent increases in angiotensin II type I receptor agonistic autoantibodies (AT1‐AA), and endothelial cell dysfunction [37]. Placental inflammation is further enhanced by this response, with subsequent release of reactive oxygen species, and pro‐inflammatory cytokines, such as TNF‐α and IL‐6, as well as the activation of cytotoxic T‐cells. These changes lead to further suppression of the Th2 cellular response and a decrease in the number of regulatory T‐cells. This causes a decrease in IL‐10 and an upregulation of pro‐inflammatory cytokines such as TNF‐α, IL‐6, IL‐12, and, ultimately, activated cytotoxic T‐cells, which further suppress anti‐inflammatory signaling [38]. Several animal model studies have shown an advantageous effect of IL‐10 administration in pregnancy‐induced hypertension [37, 38]. Furthermore, in a rat model of preeclampsia [53], animals given probiotics had lower inflammation levels and lower blood pressure compared to controls. In our study, the intervention arm of the previous preeclampsia subgroup had significantly lower levels of total lymphocytes and T‐cells at visit 2 in mid‐pregnancy compared to the placebo group. However, no differences were found in cytokine levels in stimulated and unstimulated maternal blood, which may be due to a lack of power.

Given the limitations of this study, no clinical conclusions can be drawn. However, the results may contribute to generating new hypotheses regarding the studied probiotic regimen. Interventions aimed at preventing spontaneous PTD have yielded limited success, except for prophylactic progesterone treatment in patients known to be at risk [18, 19, 54]. Prevention options for preeclampsia are limited [15], apart from prophylactic aspirin treatment for known high‐risk women [16, 17]. It is possible that *L. rhamnosus GG* might reduce the risk of spontaneous PTD by suppressing mid‐pregnancy IL‐12 levels and late‐pregnancy IL‐10 levels and reduce the risk of preeclampsia by reducing levels of total lymphocytes, T‐cells, and IL‐12, as well as increasing IL‐10 levels. However, these results should be interpreted with caution, given the limitations of the study design and outcomes. Our findings highlight the need for further basic immunological research and large‐scale randomized controlled trials to gain mechanistic insights and clinically relevant data. If future large‐scale randomized controlled trials establish a causal link between probiotic intake and a reduced risk of these pregnancy complications, prophylactic probiotic intake could constitute a low‐cost and feasible intervention.

### 4.1 Strengths and Limitations

The main strengths of this study include:

- Randomized, double‐blinded, placebo‐controlled study design.
- A substantial number of immunological parameters analyzed.
- Maternal characteristics, medical history, and detailed information about pregnancy and delivery outcomes were obtained from each participant.
- Longitudinal study design with sampling at three different time points during pregnancy, allowing investigation of the possible impact of probiotic intake timing, as well as following inflammatory changes in the normal course of pregnancy.
- The viability of the bacteria in the capsules was analyzed throughout the study, ensuring a daily dose of at least one billion viable microorganisms.
- Clearly defined exposure with a single probiotic strain is very important; the effect of different strains may vary, which necessitates analysis of individual strains.
- Information about the intake of other probiotic products was collected.
- Compliance was assessed using questionnaires and the collection of remaining capsules.

The limitations of this pilot study include the small sample size and the number of dropouts from the ITT (*n* = 15).

## 5 Conclusion

This pilot randomized placebo‐controlled trial showed that oral intake of *L. rhamnosus GG* during pregnancy did not alter TNF‐α levels in monocytes in maternal blood (primary outcome), but appeared to reduce total lymphocytes, T‐cells, IL‐10, and IL‐12 levels in mid‐pregnancy and increase IL‐10 and IL‐12 levels in late pregnancy (secondary outcomes).

## Funding

Research and Development at Södra Älvsborg Hospital (P.K., M.N.), the Swedish Society of Medicine (grant no. SLS‐961660) (P.K.), the Agreement Concerning Research and Education of Doctors (grant nos. ALFGBG‐965353, ALFGBG‐717501, ALFGBG‐426411) (B.J.), and the Health and Medical Care Committee of the Regional Executive Board, Region Västra Götaland, Sweden (grant nos. VGFOUREG‐547031, VGFOUREG‐477941) (B.J.).

## Ethics Statement

This study was conducted according to the guidelines laid down in the Declaration of Helsinki. All procedures involving human subjects were approved by the Regional Committee for Ethics in Medical Research in Gothenburg, Sweden (929‐11, 111205). All participants provided written informed consent before enrollment in the study.

## Conflicts of Interest

All authors declare: no support from any organization for the submitted paper, other than as stated under “Acknowledgements and funding” no financial relationships during the last three years with any organizations that might have an interest in this paper; no other relationships or activities that might appear to have influenced this paper. Fuko Pharma provided the *Lacticaseibacillus rhamnosus GG* and the placebo capsules but did not influence the conduct, analysis, interpretation, or final version of the paper.

## Supporting information




**Supplemental Figure 1:** Cytokine Trends in Unstimulated Maternal Blood (ITT*^†^ n* = 105).

**Supplemental Figure 2:** Cytokine Trends by Stimulation Type (ITT*^†^ n* = 105).

**Supplemental Figure 3:** IL‐12*^†^* levels in unstimulated maternal blood – at baseline and visits 2 and 3 (ITT*^‡^*, *n* = 105).

**Supplemental Figure 4:** IL‐12*^†^* levels in *L. paracasei^§^*‐stimulated maternal blood – changes over time (ITT*^‡^*, *n* = 105).

**Supplemental Figure 5:** IL‐12*^†^* levels in *P. aeruginosa^§^*‐stimulated maternal blood – at baseline and visits 2 and 3 (ITT*^‡^*, *n* = 105).

**Supplemental Figure 6:** IL‐12*^†^* levels in *P. aeruginosa^§^*‐stimulated maternal blood – changes over time (ITT*^‡^*, *n* = 105).

**Supplemenal Table 1:** Summary of Main Findings.

**Supplemental Table 2:** Clinical characteristics at the study visits.

**Supplemental Table 3:** TNF‐α*^†^* levels in maternal blood (ITT*^‡^ n* = 105) – showing no significant differences between the intervention and the placebo group.

**Supplemental Table 4a:** Subpopulations of lymphocytes (ITT, *n* = 105) – the percentage of T‐cells (T‐cell %) increase from baseline to visits 2 and 3 in both the intervention and placebo arms, where the increase from visit 2 to 3 is higher in the intervention arm.

**Supplemental Table 5a:** IL‐10^†^ levels in maternal blood (ITT, *n* = 105) – the ratio of IL‐10 levels in *Pseudomonas aeruginosa*‐stimulated and unstimulated maternal blood increases more from visit 2 to visit 3 in the intervention arm than the placebo arm.

**Supplemental Table 6a:** IL‐12^†^ levels in maternal blood (ITT*^‡^*, *n* = 105) – showing that IL12 levels in both unstimulated maternal blood and *E. coli* LPS stimulated maternal blood decrease from baseline to visit 2 in the intervention arm and increase in the placebo arm. On the contrary, IL‐12 levels in *P. aeruginosa‐*stimulated maternal blood increased from visit 2 to 3 in the intervention arm and increased in the placebo arm.

**Supplemental File 1:** aji70190‐sup‐0013‐SuppMat.docx

**Supplemental File 2**: aji70190‐sup‐0014‐SuppMat.docx

## Data Availability

The data that support the findings of this study are available on request from the corresponding author. The data are not publicly available due to privacy or ethical restrictions.
