# Supplementary material for: Oral Lacticaseibacillus rhamnosus GG Exposure During Pregnancy and Effects on Maternal Inflammatory Response—A Blinded, Pilot Randomized, Placebo‐Controlled Study
Source: Am J Reprod Immunol. 2025 Dec 10;94(6):e70190. doi: 10.1111/aji.70190 (PMC12692997; doi:10.1111/aji.70190)
Supplement: Supplementary file 1 — Supplemental Figure 1: Cytokine Trends in Unstimulated Maternal Blood (ITT † n = 105). [file AJI-94-e70190-s003.docx]

**Supplemental Figure 1. Cytokine Trends in Unstimulated Maternal Blood (ITT***^†^* **n=105)**


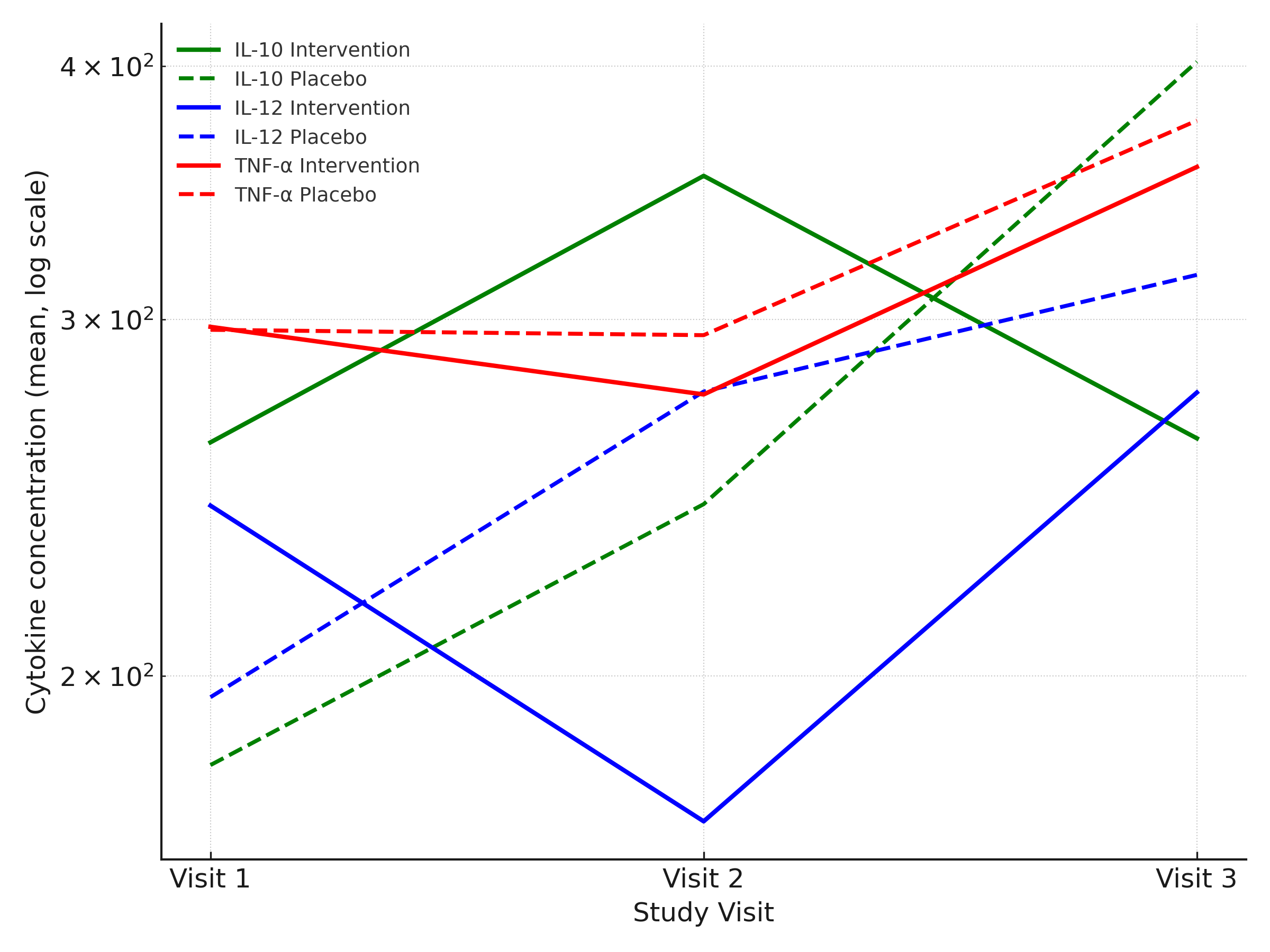


Mean cytokine levels (IL-10, IL-12, TNF-α) in unstimulated maternal blood at Visits 1–3. Data derived from Supplemental Tables 2, 4b, and 5a.

*† Intention to treat*
