# Supplementary material for: Oral Lacticaseibacillus rhamnosus GG Exposure During Pregnancy and Effects on Maternal Inflammatory Response—A Blinded, Pilot Randomized, Placebo‐Controlled Study
Source: Am J Reprod Immunol. 2025 Dec 10;94(6):e70190. doi: 10.1111/aji.70190 (PMC12692997; doi:10.1111/aji.70190)
Supplement: Supplementary file 2 — Supplemental Figure 2: Cytokine Trends by Stimulation Type (ITT † n = 105). [file AJI-94-e70190-s010.docx]

# Supplemental Figure 2. Cytokine Trends by Stimulation Type (ITT*^†^* n=105)


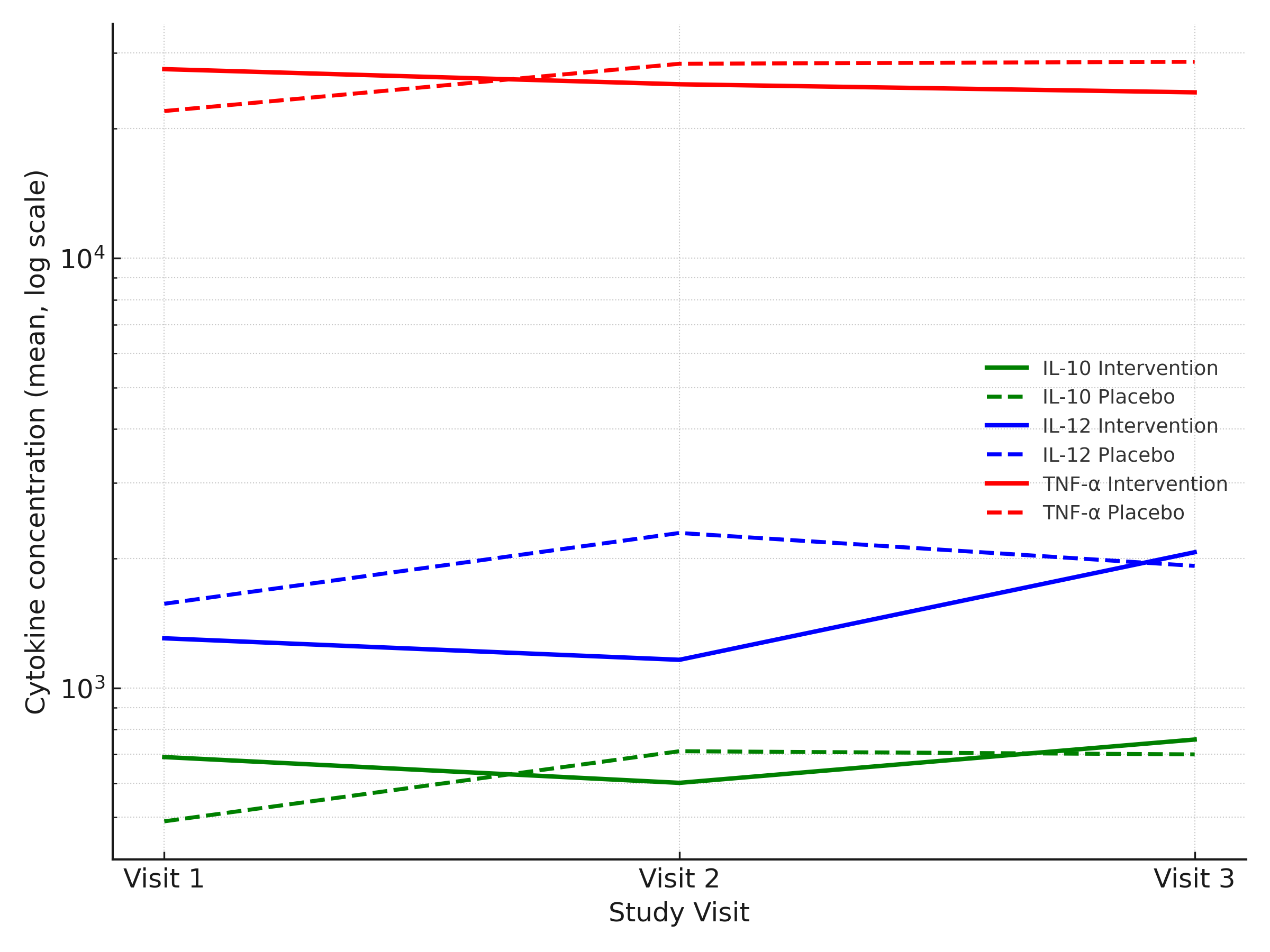


Mean cytokine levels in maternal blood stimulated with *Pseudomonas aeruginosa* (IL-10, IL-12) and *E. coli* LPS (TNF-α) at Visits 1–3. Data derived from Supplemental Tables 2, 4a, and 5a.

*† Intention to treat*
