# Supplementary material for: Oral Lacticaseibacillus rhamnosus GG Exposure During Pregnancy and Effects on Maternal Inflammatory Response—A Blinded, Pilot Randomized, Placebo‐Controlled Study
Source: Am J Reprod Immunol. 2025 Dec 10;94(6):e70190. doi: 10.1111/aji.70190 (PMC12692997; doi:10.1111/aji.70190)
Supplement: Supplementary file 3 — Supplemental Figure 3: IL‐12 † levels in unstimulated maternal blood – at baseline and visits 2 and 3 (ITT ‡ , n = 105). [file AJI-94-e70190-s002.docx]

**Supplemental Figure 3. IL-12***^†^* **levels in unstimulated maternal blood – at baseline and visits 2 and 3 (ITT***^‡^***, n=105).**


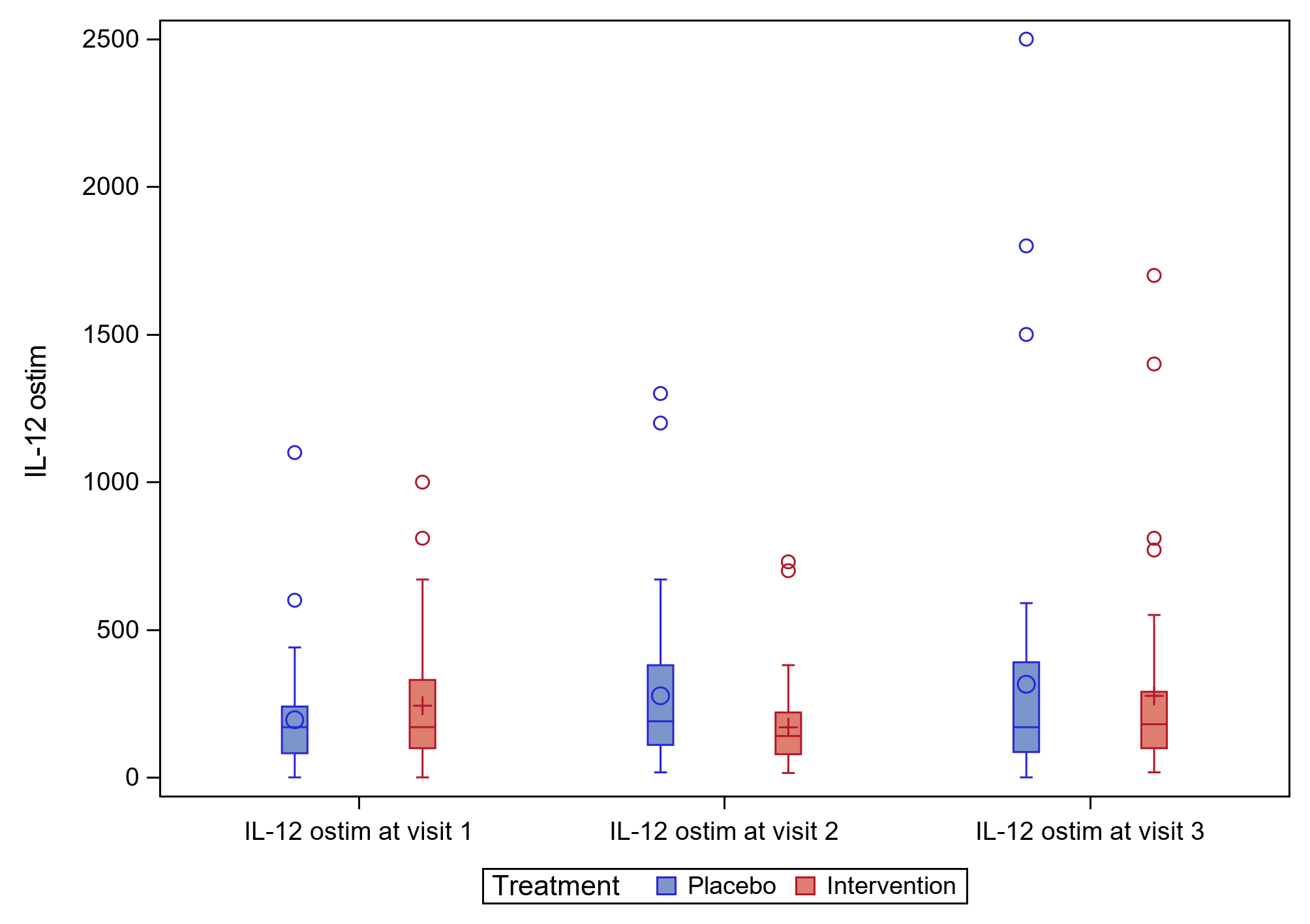


Boxplot diagram of IL-12 levels in unstimulated maternal blood, illustrating lower levels at visit 2 in the intervention arm (p=0.008). Analyses were performed in the ITT population. The box represents Q1 to Q3. The vertical line in the box is the median. The circle (in the placebo arm) or the plus sign (in the intervention arm) represents the mean. The whiskers represent the 1.5 interquartile range from Q1 and Q3, values above or below are shown as individual circles.

*†* Interleukin-12

*‡* Intention-to-treat

baseline

visit 3

visit 2

IL-12 in unstimulated maternal blood,

cytokine-positive monocytes/ml
