# Supplementary material for: Oral Lacticaseibacillus rhamnosus GG Exposure During Pregnancy and Effects on Maternal Inflammatory Response—A Blinded, Pilot Randomized, Placebo‐Controlled Study
Source: Am J Reprod Immunol. 2025 Dec 10;94(6):e70190. doi: 10.1111/aji.70190 (PMC12692997; doi:10.1111/aji.70190)
Supplement: Supplementary file 7 — Supplemenal Table 1: Summary of Main Findings. [file AJI-94-e70190-s001.docx]

**Supplemenal Table 1. Summary of Main Findings**

| **Outcome / Marker** | **Time Point** | **Intervention Effect (L. rhamnosus GG vs Placebo)** | **p-value / Significance** | **Comment / Interpretation** |
| --- | --- | --- | --- | --- |
| TNF-α (Primary outcome) | Visit 2–3 | No significant difference in ITT; lower TNF-α in sensitivity analysis (Δ=−11785 cells/mL) | p=0.03 (sensitivity) | Reduced TNF-α when excluding fever/antibiotics |
| Total lymphocytes | Visit 2 | Lower levels in intervention group | Exploratory | Notable in previous preeclampsia subgroup |
| T-cells (total) | Visit 2 | Lower in intervention group | Exploratory | Suggests dampened immune activation |
| Helper T-cells (CD4+) | Visits 1–3 | Lower across all visits (preeclampsia subgroup) | Exploratory | Possible anti-inflammatory trend |
| Regulatory T-cells (%) | Visit 3 | Decreased in intervention group (control subgroup) | Exploratory | Opposite direction compared to placebo |
| IL-10 (anti-inflammatory) | Visit 2 → 3 | Increased in intervention group (P. aeruginosa stimulation) | p=0.024 | Enhanced anti-inflammatory response late in pregnancy |
| IL-12 (pro-inflammatory) | Visit 2 | Lower in intervention group (LPS, L. paracasei, P. aeruginosa stimulation) | p=0.014–0.043 | Reduced pro-inflammatory activity mid-pregnancy |
| IL-12 | Visit 3 | Increased in intervention group (P. aeruginosa stimulation) | p=0.044 | Normalization in late pregnancy |
