# Supplementary material for: Oral Lacticaseibacillus rhamnosus GG Exposure During Pregnancy and Effects on Maternal Inflammatory Response—A Blinded, Pilot Randomized, Placebo‐Controlled Study
Source: Am J Reprod Immunol. 2025 Dec 10;94(6):e70190. doi: 10.1111/aji.70190 (PMC12692997; doi:10.1111/aji.70190)
Supplement: Supplementary file 8 — Supplemental Table 2: Clinical characteristics at the study visits. [file AJI-94-e70190-s006.docx]

### Supplemental Table 2. Clinical characteristics at the study visits.

| Variable | Intervention (n=53) | Placebo (n=52) | p-value | Difference between arms Mean (95% CI) |
| --- | --- | --- | --- | --- |
| Gestational age (days), baseline | 98.0 (17.9) 100 (46; 123) (88; 112) n=53 | 99.5 (18.0) 102 (57; 128) (85.5; 116) n=52 | 0.67 | -1.52 (-8.46; 5.42) |
| Gestational age (days), visit 2 | 174.9 (5.7) 176 (158; 188) (172; 178) n=53 | 176.9 (7.6) 177 (160; 198) (171; 181) n=52 | 0.14 | -1.98 (-4.59; 0.64) |
| Gestational age (days), visit 3 | 246.4 (4.7) 246 (237; 263) (244; 249) n=47 | 247.6 (5.7) 248 (239; 266) (245; 251) n=47 | 0.26 | -1.21 (-3.34; 0.92) |
| Other probiotic intake, baseline | 24 (45.3%) | 23 (44.2%) | 1.00 | 1.1 (-19.9; 22.0) |
| Other probiotic intake, visit 2 | 9 (17.0%) | 6 (11.5%) | 0.61 | 5.4 (-9.8; 20.7) |
| Other probiotic intake, visit 3 | 4 (7.7%) | 6 (12.0%) | 0.69 | -4.3 (-17.8; 9.2) |
| Iron supplement intake, baseline | 18 (34.6%) | 23 (44.2%) | 0.42 | -9.6 (-30.2; 11.0) |
| Iron supplement intake, visit 2 | 42 (79.2%) | 35 (68.6%) | 0.31 | 10.6 (-8.1; 29.3) |
| Iron supplement intake, visit 3 | 34 (73.9%) | 38 (82.6%) | 0.45 | -8.7 (-27.6; 10.2) |
| Antibiotics < 1 week before baseline | 0 (0.0%) | <3 | 1.00 | -2.0 (-7.7; 3.8) |
| Antibiotics < 1 week before visit 2 | <3 | 0 (0.0%) | 0.50 | 3.8 (-3.3; 10.8) |
| Antibiotics < 1 week before visit 3 | <3 | 0 (0.0%) | 1.00 | 1.9 (-3.7; 7.5) |
| Fever 1 <week before baseline | 8 (15.1%) | 9 (17.3%) | 0.97 | -2.2 (-18.2; 13.8) |
| Fever 1 <week before visit 2 | 5 (9.4%) | 8 (15.7%) | 0.51 | -6.3 (-20.9; 8.4) |
| Fever 1 <week before visit 3 | 9 (17.0%) | 6 (11.5%) | 0.61 | 5.4 (-9.8; 20.7) |

Analysis was performed on the intention-to-treat population (n=105). For categorical variables, n (%) is presented. For continuous variables, mean (SD) / median (min; max) / (Q1; Q3) / n are presented. For comparison between arms, Fisher´s exact test (lowest 1-sided p value multiplied by 2) was used for dichotomous variables and Student’s t-test was used for continuous variables. The confidence intervals for dichotomous variables correspond to the unconditional exact confidence limits. If no exact limits could be computed, asymptotic Wald confidence limits with
continuity correction were calculated instead. The calculation of confidence intervals for continuous variables is based on the assumption of
normality. When variances are not equal (p<0.05), the SD is based on Satterthwaite´s approximation; otherwise, the SD is based on the pooled SDs.

Variables where data were available for fewer than 3 individuals are reported as ‘<3’ in order to protect privacy.
