# Supplementary material for: Oral Lacticaseibacillus rhamnosus GG Exposure During Pregnancy and Effects on Maternal Inflammatory Response—A Blinded, Pilot Randomized, Placebo‐Controlled Study
Source: Am J Reprod Immunol. 2025 Dec 10;94(6):e70190. doi: 10.1111/aji.70190 (PMC12692997; doi:10.1111/aji.70190)
Supplement: Supplementary file 9 — Supplemental Table 3: TNF‐α † levels in maternal blood (ITT ‡ n = 105) – showing no significant differences between the intervention and the placebo group. [file AJI-94-e70190-s008.docx]

Supplemental Table 3. TNF-α*^†^* levels in maternal blood (ITT*^‡^* n=105) – showing no significant differences between the intervention and the placebo group.

|  | Intervention (n=53) | | Placebo (n=52) | |  | |
| --- | --- | --- | --- | --- | --- | --- |
| Variable | Mean (SD) Median (Min; Max) (Q1; Q3) n | p-value within arm | Mean (SD) Median (Min; Max) (Q1; Q3) n | p-value within arm | p-value between arms | Difference between arms Mean (95% CI) |
| TNF-α*^†^* in unstimulated maternal blood at baseline  (number of cytokine-positive monocytes/ml) | 323.7 (301.8) 250 (18; 1600) (125; 390) n=52 |  | 282.9 (283.2) 220 (0; 1800) (130; 360) n=51 |  | 0.49 | 40.8 (-72.4; 153.9) |
| TNF-α in unstimulated maternal blood at visit 2  (number of cytokine-positive monocytes/ml) | 239.2 (175.7) 200 (15; 980) (130; 285) n=52 |  | 310.1 (286.0) 220 (36; 1500) (93; 450) n=51 |  | 0.13 | -70.9 (-163.2; 19.0) |
| TNF-α in unstimulated maternal blood at visit 3  (number of cytokine-positive monocytes/ml) | 320.1 (371.4) 240 (0; 2400) (120; 360) n=47 |  | 328.7 (437.9) 180 (0; 2600) (110; 360) n=46 |  | 0.92 | -8.52 (-171.59; 158.75) |
| TNF-α in unstimulated maternal blood – change from baseline to visit 2  (number of cytokine-positive monocytes/ml) | -59.6 (306.3) -50 (-910; 912) (-190; 100) n=51 | 0.17 | 23.0 (376.1) 14.5 (-1320; 1030) (-180; 220) n=50 | 0.67 | 0.23 | -82.6 (-216.6; 50.9) |
| TNF-α in unstimulated maternal blood – change from baseline to visit 3  (number of cytokine-positive monocytes/ml) | 28.2 (463.2) -6 (-890; 2332) (-170; 156) n=46 | 0.73 | 50.9 (464.0) -20 (-890; 2360) (-149; 61) n=45 | 0.52 | 0.82 | -22.7 (-209.7; 168.7) |
| TNF-α in unstimulated maternal blood – change from visit 2 to visit 3  (number of cytokine-positive monocytes/ml) | 73.3 (286.5) 50 (-360; 1420) (-100; 160) n=47 | 0.079 | 54.0 (519.0) 1 (-812; 2540) (-150; 118) n=45 | 0.53 | 0.83 | 19.3 (-149.0; 188.1) |
| TNF-α in *Escherichia coli* LPS*^§^*-stimulated maternal blood at baseline  (number of cytokine-positive monocytes/ml) | 28676 (22400) 22000 (4000; 110000) (15000; 35000) n=51 |  | 23312 (16409) 18000 (3000; 74000) (12000; 27000) n=51 |  | 0.17 | 5365 (-2262; 12995) |
| TNF-α in *E. coli* LPS-stimulated maternal blood at visit 2  (number of cytokine-positive monocytes/ml) | 24336 (15734) 21000 (650; 72000) (13500; 31500) n=52 |  | 24973 (21735) 18000 (2200; 100000) (9700; 36000) n=51 |  | 0.86 | -637 (-7967; 6820) |
| TNF-α in *E. coli* LPS-stimulated maternal blood at visit 3  (number of cytokine-positive monocytes/ml) | 26853 (14148) 26000 (690; 73000) (17000; 33000) n=47 |  | 26280 (23266) 21500 (1800; 150000) (12000; 32000) n=46 |  | 0.90 | 573 (-7148; 8161) |
| TNF-α in *E. coli* LPS-stimulated maternal blood – change from baseline to visit 2  (number of cytokine-positive monocytes/ml) | -4481 (19434) -2450 (-61000; 38000) (-15000; 7000) n=50 | 0.11 | 2514 (23709) 250 (-54600; 81000) (-9000; 11000) n=50 | 0.46 | 0.11 | -6995 (-15654; 1759) |
| TNF-α in *E. coli* LPS-stimulated maternal blood – change from baseline to visit 3  (number of cytokine-positive monocytes/ml) | -1571 (23136) 2000 (-81000; 42000) (-8000; 12000) n=45 | 0.65 | 3633 (22594) 2000 (-47000; 116000) (-4000; 11000) n=45 | 0.33 | 0.29 | -5205 (-14690; 4305) |
| TNF-α in *E. coli* LPS-stimulated maternal blood – change from visit 2 to visit 3  (number of cytokine-positive monocytes/ml) | 2567 (18112) 3000 (-44000; 39000) (-11900; 13600) n=47 | 0.34 | -653 (19230) -2000 (-60000; 50000) (-8000; 10000) n=45 | 0.83 | 0.41 | 3220 (-4433; 10945) |
| Difference in TNF-α in *E. coli* LPS-stimulated and unstimulated maternal blood at baseline  (number of cytokine-positive monocytes/ml) | 28348 (22313) 21820 (3981; 109470) (14690; 34620) n=51 |  | 23029 (16304) 17750 (2979; 73320) (11730; 26690) n=51 |  | 0.17 | 5319 (-2268; 12914) |
| Difference in TNF-α in *E. coli* LPS-stimulated and unstimulated maternal blood at visit 2  (number of cytokine-positive monocytes/ml) | 24096 (15730) 20405 (430; 71800) (13397; 31360) n=52 |  | 24662 (21652) 17620 (1830; 99870) (9664; 35490) n=51 |  | 0.88 | -566 (-7894; 6878) |
| Difference in TNF-α in *E. coli* LPS-stimulated and unstimulated maternal blood at visit 3  (number of cytokine-positive monocytes/ml) | 26533 (14161) 25901 (470; 72690) (16400; 32620) n=47 |  | 25952 (23111) 21389 (1750; 148500) (11952; 31750) n=46 |  | 0.90 | 581 (-7119; 8140) |
| Difference in TNF-α in *E. coli* LPS-stimulated and unstimulated maternal blood – change from baseline to visit 2  (number of cytokine-positive monocytes/ml) | -4402 (19316) -2372 (-60620; 37940) (-14820; 6798) n=50 | 0.11 | 2491 (23677) 187 (-54521; 80760) (-8840; 10912) n=50 | 0.46 | 0.12 | -6893 (-15524; 1840) |
| Difference in TNF-α in *E. coli* LPS-stimulated and unstimulated maternal blood – change from baseline to visit 3  (number of cytokine-positive monocytes/ml) | -1548 (23048) 1730 (-80600; 42560) (-8280; 11850) n=45 | 0.65 | 3582 (22460) 1932 (-46922; 114730) (-3780; 10980) n=45 | 0.33 | 0.29 | -5131 (-14588; 4329) |
| Difference in TNF-α in *E. coli* LPS-stimulated and unstimulated maternal blood – change from visit 2 to visit 3  (number of cytokine-positive monocytes/ml) | 2493 (18072) 2978 (-44150; 38775) (-11669; 13430) n=47 | 0.35 | -707 (19097) -2070 (-59930; 48630) (-8085; 10050) n=45 | 0.81 | 0.41 | 3201 (-4410; 10882) |
| Ratio of TNF-α in *E. coli* LPS-stimulated and unstimulated maternal blood at baseline | 157.0 (164.9) 93.1 (16.3; 833.3) (53.6; 200) n=51 |  | 123.7 (122.0) 89 (27.8; 680) (54.5; 145.3) n=50 |  | 0.26 | 33.3 (-23.6; 90.3) |
| Ratio of TNF-α in *E. coli* LPS-stimulated and unstimulated maternal blood at visit 2 | 171.6 (216.3) 100 (3; 1133.3) (59.7; 214.9) n=52 |  | 138.5 (151.6) 92.6 (5.9; 769.2) (45; 166.7) n=51 |  | 0.38 | 33.1 (-38.6; 105.3) |
| Ratio of TNF-α in *E. coli* LPS-stimulated and unstimulated maternal blood at visit 3 | 143.1 (120.1) 115.3 (3.1; 603.4) (63; 177.8) n=46 |  | 130.4 (94.1) 115.2 (3.4; 426.2) (70.7; 164.7) n=45 |  | 0.59 | 12.6 (-32.2; 56.8) |
| Ratio of TNF-α in *E. coli* LPS-stimulated and unstimulated maternal blood – change from baseline to visit 2 | 15.2 (256.5) 7.7 (-733.3; 978.4) (-77.7; 111.9) n=50 | 0.68 | 18.4 (193.4) 11.1 (-632.6; 621.4) (-48.9; 97.7) n=49 | 0.51 | 0.95 | -3.20 (-92.57; 86.97) |
| Ratio of TNF-α in *E. coli* LPS-stimulated and unstimulated maternal blood – change from baseline to visit 3 | -7.51 (185.44) -3.27 (-525.27; 550.11) (-87.43; 87.72) n=44 | 0.79 | 15.0 (99.1) 22.7 (-253.7; 340.5) (-41.7; 63.6) n=43 | 0.33 | 0.49 | -22.5 (-85.8; 40.2) |
| Ratio of TNF-α in *E. coli* LPS-stimulated and unstimulated maternal blood – change from visit 2 to visit 3 | -31.5 (246.1) 8.9 (-984.8; 526.3) (-62.3; 61.9) n=46 | 0.41 | -22.9 (190.1) -1.7 (-669.2; 333.6) (-79.9; 66.7) n=44 | 0.43 | 0.86 | -8.55 (-100.95; 82.14) |
| Analysis performed on the intention-to-treat (ITT) population. For continuous variables, mean (SD) / median (min; max) / (Q1; Q3) / n are presented. For comparison between arms, Fisher’s non-parametric permutation test was used for continuous variables. For comparison within arms, Fisher’s non-parametric permutation test for matched pairs was used. The confidence interval for the mean difference between arms is based on Fisher’s non-parametric permutation test.  *†* Tumor necrosis factor-alpha  *‡* Intention-to-treat  *§* Lipopolysaccharide from *Escherichia coli* | | | | | | |
