# Supplementary material for: Oral Lacticaseibacillus rhamnosus GG Exposure During Pregnancy and Effects on Maternal Inflammatory Response—A Blinded, Pilot Randomized, Placebo‐Controlled Study
Source: Am J Reprod Immunol. 2025 Dec 10;94(6):e70190. doi: 10.1111/aji.70190 (PMC12692997; doi:10.1111/aji.70190)
Supplement: Supplementary file 10 — Supplemental Table 4a: Subpopulations of lymphocytes (ITT, n = 105) – the percentage of T‐cells (T‐cell %) increase from baseline to visits 2 and 3 in both the intervention and placebo arms, where the increase from visit 2 to 3 is higher in the intervention arm. [file AJI-94-e70190-s005.docx]

**Supplemental Table 4a. Subpopulations of lymphocytes (ITT, n=105) – the percentage of T-cells (T-cell %) increase from baseline to visits 2 and 3 in both the intervention and placebo arms, where the increase from visit 2 to 3 is higher in the intervention arm.**

|  | **Intervention (n=53)** | | **Placebo (n=52)** | |  | |
| --- | --- | --- | --- | --- | --- | --- |
| **Variable** | **Mean (SD) Median (Min; Max) (Q1; Q3)** | **p-value within arm** | **Mean (SD) Median (Min; Max) (Q1; Q3)** | **p-value within arm** | **p-value between arms** | **Difference between arms Mean (95% CI)** |
| **T-cells % in maternal blood at baseline** | 78.2 (5.4) 79 (60; 87) (76; 82) n=53 |  | 79.0 (4.9) 79 (70; 88) (74; 83) n=51 |  | 0.46 | -0.794 (-2.800; 1.190) |
| **T-cells % in maternal blood at visit 2** | 79.4 (5.8) 80.5 (61; 88) (76; 83) n=52 |  | 80.2 (4.5) 81 (72; 90) (77; 83) n=52 |  | 0.47 | -0.769 (-2.800; 1.240) |
| **T-cells % in maternal blood at visit 3** | 81.4 (5.3) 82 (64; 90) (79; 85) n=47 |  | 81.4 (4.6) 82 (70; 90) (78; 85) n=47 |  | 1.00 | 0.021 (-2.038; 2.050) |
| **T-cells % in maternal blood – change from visit 2 to visit 3** | 2.19 (1.75) 2 (-1; 7) (1; 3) n=47 | <.0001 | 1.43 (1.81) 1 (-2; 5) (0; 3) n=47 | <.0001 | 0.047 | 0.766 (0.042; 1.500) |
| Analysis performed on the intention-to-treat (ITT) population. For continuous variables, mean (SD) / median (min; max) / (Q1; Q3) / n are presented. For comparison between arms, Fisher’s non parametric permutation test was used for continuous variables. For comparison within arms, Fisher’s non-parametric permutation test for matched pairs was used. The confidence interval for the mean difference between arms is based on Fisher’s non-parametric permutation test. | | | | | | |

**Supplemental Table 4b. Subpopulations of lymphocytes in maternal blood, in previous preeclampsia subgroup (n=35) – levels of lymphocytes and absolute levels of T-cells are lower at visit 2 and levels of T-helper cells are lower at all three visits in the intervention arm than the placebo arm.**

|  | **Intervention (n=18)** | | | **Placebo (n=17)** | | |  | | |
| --- | --- | --- | --- | --- | --- | --- | --- | --- | --- |
| **Variable** | **Mean (SD) Median (Min; Max) (Q1; Q3) n=** | **Adjusted means**† **SEM (95% CI)** | **p-value within arm** | **Mean (SD) Median (Min; Max) (Q1; Q3) n=** | **Adjusted means**† **SEM (95% CI)** | **p-value within arm** | **p-value between arms** | **Adjusted p-value**† | **Difference between arms Adjusted means (95% CI)** |
| **Lymphocytes in maternal blood at visit 2**  **number (x 10^9^/L) of lymphocytes** | 1.42 (0.34) 1.3 (0.91; 2.1) (1.2; 1.7) n=17 | 1.40 0.08 (1.24-1.56) |  | 1.69 (0.38) 1.7 (1.2; 2.4) (1.3; 1.8) n=17 | 1.71 0.08 (1.55-1.87) |  | 0.043 | 0.010 | -0.309 (-0.539; -0.078) |
| **T-cells (absolute) in maternal blood at visit 2**  **number (x 10^9^/L) of lymphocytes** | 1.13 (0.27) 1.1 (0.73; 1.7) (0.91; 1.2) n=17 | 1.11 0.07 (0.97-1.24) |  | 1.35 (0.33) 1.3 (0.95; 2) (1.1; 1.5) n=17 | 1.36 0.07 (1.23-1.50) |  | 0.040 | 0.011 | -0.256 (-0.448; -0.064) |
| **T-helper cells (absolute) in maternal blood at baseline**  **number (x 10^9^/L) of lymphocytes** | 0.694 (0.159) 0.65 (0.48; 0.99) (0.58; 0.86) n=18 | 0.686 0.051 (0.583-0.790) |  | 0.829 (0.279) 0.77 (0.49; 1.4) (0.59; 1) n=17 | 0.838 0.052 (0.732-0.944) |  | 0.093 | 0.046 | -0.151 (-0.300; -0.003) |
| **T-helper cells (absolute) in maternal blood at visit 2**  **number (x 10^9^/L) of lymphocytes** | 0.675 (0.179) 0.62 (0.44; 0.98) (0.52; 0.8) n=17 | 0.664 0.053 (0.555-0.773) |  | 0.844 (0.276) 0.81 (0.52; 1.4) (0.59; 0.98) n=17 | 0.855 0.053 (0.746-0.964) |  | 0.043 | 0.017 | -0.191 (-0.346; -0.036) |
| **T-helper cells (absolute) in maternal blood at visit 3**  **number (x 10^9^/L) of lymphocytes** | 0.730 (0.166) 0.74 (0.4; 1.1) (0.65; 0.81) n=17 | 0.719 0.049 (0.619-0.819) |  | 0.852 (0.256) 0.82 (0.49; 1.4) (0.63; 1) n=17 | 0.863 0.049 (0.763-0.964) |  | 0.11 | 0.048 | -0.144 (-0.287; -0.002) |
| Analysis performed on the intention-to-treat (ITT) population. For continuous variables, mean (SD) / median (min; max) / (Q1; Q3) / n are presented. For comparison between arms, Student’s t-test was used for continuous variables. For comparison within arms, paired Student´s t-test was used.  † Adjusting for maternal body mass index (BMI) using Analysis of Covariance (ANCOVA). | | | | | | | | | |

**Supplemental Table 4c. Subpopulations of lymphocytes in maternal blood, in the control subgroup (n=33) – the percentage of regulatory T-cells (Treg %) is lower at visit 3 in the intervention arm than the placebo arm and decreases from visit 2 to visit 3 in the intervention arm.**

|  | **Intervention (n=17)** | | | **Placebo (n=16)** | | |  | | |
| --- | --- | --- | --- | --- | --- | --- | --- | --- | --- |
| **Variable** | **Mean (SD) Median (Min; Max) (Q1; Q3)** | **Adjusted means**† **SEM (95% CI)** | **p-value within arm** | **Mean (SD) Median (Min; Max) (Q1; Q3)** | **Adjusted means**† **SEM (95% CI)** | **p-value within arm** | **p-value between arms** | **Adjusted p-value**† | **Difference between arms Adjusted means (95% CI)** |
| **Treg***^‡^* **% lymphocytes in maternal blood at visit 3**  **number (x 109/L) of lymphocytes** | 0.490 (0.267) 0.48 (0.19; 1.2) (0.305; 0.525) n=16 | 0.463 0.083 (0.293-0.633) |  | 0.702 (0.368) 0.67 (0.17; 1.3) (0.45; 0.86) n=15 | 0.731 0.086 (0.555-0.907) |  | 0.075 | 0.040 | -0.267 (-0.522; -0.013) |
| **Treg % lymphocytes in maternal blood – change from visit 2 to visit 3**  **number (x 10^9^/L) of lymphocytes** | -0.192 (0.294) -0.175 (-0.73; 0.48) (-0.435; -0.015) n=16 | -0.247 0.100 (-0.452--0.042) | 0.020 | 0.100 (0.491) 0.18 (-0.78; 0.94) (-0.26; 0.28) n=15 | 0.159 0.104 (-0.053-0.371) | 0.44 | 0.052 | 0.011 | -0.406 (-0.713; -0.099) |
| Analysis performed on the intention-to-treat (ITT) population. For continuous variables, mean (SD) / median (min; max) / (Q1; Q3) / n are presented. For comparison between arms, Student’s t-test was used for continuous variables. For comparison within arms, paired Student´s t-test was used.  †Adjusting for maternal body mass index (BMI) using Analysis of Covariance (ANCOVA).  *‡* Regulatory T-cells | | | | | | | | | |
