# Supplementary material for: Oral Lacticaseibacillus rhamnosus GG Exposure During Pregnancy and Effects on Maternal Inflammatory Response—A Blinded, Pilot Randomized, Placebo‐Controlled Study
Source: Am J Reprod Immunol. 2025 Dec 10;94(6):e70190. doi: 10.1111/aji.70190 (PMC12692997; doi:10.1111/aji.70190)
Supplement: Supplementary file 11 — Supplemental Table 5a: IL‐10† levels in maternal blood (ITT, n = 105) – the ratio of IL‐10 levels in Pseudomonas aeruginosa‐stimulated and unstimulated maternal blood increases more from visit 2 to visit 3 in the intervention arm than the placebo arm. [file AJI-94-e70190-s013.docx]

|  | Intervention (n=53) | | Placebo (n=52) | |  | |
| --- | --- | --- | --- | --- | --- | --- |
| Variable | Mean (SD) Median (Min; Max) (Q1; Q3) | p-value within arm | Mean (SD) Median (Min; Max) (Q1; Q3) | p-value within arm | p-value between arms | Difference between arms Mean (95% CI) |
| IL-10 in *P. aeruginosa*-stimulated maternal blood at baseline  (number of cytokine-positive monocytes/ml) | 690 (906) 400 (61; 4800) (240; 660) n=51 |  | 489.4 (378.9) 420 (0; 1600) (220; 720) n=50 |  | 0.16 | 200.9 (-67.4; 473.0) |
| IL-10 in *P. aeruginosa*-stimulated maternal blood at visit 2  (number of cytokine-positive monocytes/ml) | 601 (547) 370 (0; 2300) (210; 910) n=51 |  | 712 (723) 430 (0; 3300) (220; 970) n=51 |  | 0.38 | -110.2 (-363.5; 141.1) |
| IL-10 in *P. aeruginosa*-stimulated maternal blood at visit 3  (number of cytokine-positive monocytes/ml) | 758 (701) 600 (17; 3400) (210; 1100) n=47 |  | 700 (628) 585 (0; 3000) (290; 790) n=46 |  | 0.68 | 58.5 (-217.3; 329.6) |
| Ratio of IL-10 in *P. aeruginosa*-stimulated and unstimulated maternal blood – change from visit 2 to visit 3 | 2.91 (10.79) 1 (-9.53; 63.89) (-1.06; 4.09) n=45 | 0.028 | -1.23 (7.16) -0.08 (-19; 15.79) (-3.49; 1.41) n=42 | 0.27 | 0.024 | 4.14 (0.45; 7.98) |
| Analysis performed on the intention-to-treat (ITT) population. For continuous variables, mean (SD) / median (min; max) / (Q1; Q3) / n are presented. For comparison between arms, Fisher’s non-parametric permutation test was used for continuous variables. For comparison within arms, Fisher’s non- parametric permutation test for matched pairs was used. The confidence interval for the mean difference between arms is based on Fisher’s non-parametric permutation test.  † Interleukin-10 | | | | | | |

Supplemental Table 5a. IL-10^†^ levels in maternal blood (ITT, n=105) – the ratio of IL-10 levels in *Pseudomonas aeruginosa*-stimulated and unstimulated maternal blood increases more from visit 2 to visit 3 in the intervention arm than the placebo arm.

Supplemental Table 5b. IL-10^†^ levels in maternal blood in the previous preterm delivery subgroup (n=37) – levels in unstimulated maternal blood decrease from visit 2 to 3 in the intervention arm and increase in the placebo arm; the difference in and the ratio of IL-10 levels in *Pseudomonas aeruginosa*-stimulated and unstimulated maternal blood decrease from visit 1 to 2 in the intervention arm and increase in the placebo arm.

|  | Intervention (n=18) | | | Placebo (n=19) | | |  | | |
| --- | --- | --- | --- | --- | --- | --- | --- | --- | --- |
| Variable | Mean (SD) Median (Min; Max) (Q1; Q3) | Adjusted means† SEM (95% CI) | p-value within arm | Mean (SD) Median (Min; Max) (Q1; Q3) | Adjusted means^‡^ SEM (95% CI) | p-value within arm | p-value between arms | Adjusted p-value^‡^ | Difference between arms Adjusted means (95% CI) |
| IL-10 in unstimulated maternal blood at baseline  (number of cytokine-positive monocytes/ml) | 260.8 (215.1) 200 (19; 730) (93; 420) n=18 | 226.4 45.0 (134.8-318.0) |  | 180.7 (166.1) 120 (0; 640) (80; 250) n=18 | 187.2 43.6 (98.4-276.0) |  | 0.22 | 0.55 | 39.2 (-93.3; 171.7) |
| IL-10 in unstimulated maternal blood at visit 2  (number of cytokine-positive monocytes/ml) | 353.1 (393.0) 200 (53; 1400) (93; 380) n=18 | 383.7 80.3 (220.2-547.1) |  | 243.1 (213.4) 210 (0; 900) (80; 330) n=19 | 230.8 75.7 (76.8-384.7) |  | 0.30 | 0.19 | 152.9 (-80.2; 386.0) |
| IL-10 in unstimulated maternal blood at visit 3  (number of cytokine-positive monocytes/ml) | 262.0 (241.4) 185 (17; 720) (62; 360) n=14 | 228.9 145.6 (-71.0-528.9) |  | 402.0 (659.5) 180 (0; 2600) (88; 480) n=15 | 444.8 134.9 (166.9-722.7) |  | 0.45 | 0.30 | -215.8 (-638.3; 206.7) |
| IL-10 in unstimulated maternal blood – change from visit 2 to visit 3  (number of cytokine-positive monocytes/ml) | -152.9 (404.4) -73 (-1090; 440) (-393; 74) n=14 | -229.7 140.5 (-519.1-59.8) | 0.18 | 195.9 (571.7) 49 (-240; 2020) (-168; 260) n=15 | 253.2 130.2 (-14.9-521.4) | 0.21 | 0.070 | 0.022 | -482.9 (-890.6; -75.2) |
| IL-10 in *P. aeruginosa*-stimulate maternal blood at baseline  (number of cytokine-positive monocytes/ml) | 870 (1203) 400 (73; 4800) (240; 910) n=18 | 845 208 (421-1268) |  | 351.4 (279.6) 360 (0; 980) (120; 510) n=17 | 275.1 207.8 (-148.6-698.8) |  | 0.091 | 0.070 | 569 (-49; 1188) |
| IL-10 in *P. aeruginosa*-stimulate maternal blood at visit 2  (number of cytokine-positive monocytes/ml) | 557 (589) 340 (0; 2300) (110; 910) n=17 | 514 200 (107-921) |  | 746 (866) 280 (0; 3300) (170; 1000) n=19 | 764 182 (393-1135) |  | 0.45 | 0.38 | -250.1 (-820.8; 320.6) |
| Difference in IL-10 in *P. aeruginosa*-stimulated and unstimulated maternal blood – change from baseline to visit 2  (number of cytokine-positive monocytes/ml) | -387.0 (1344.3) 54 (-4927; 1480) (-440; 173) n=17 | -457.3 297.5 (-1065-150.4) | 0.25 | 383.7 (944.9) 121 (-950; 2915) (-30; 864) n=17 | 487.4 288.1 (-101.0-1075.8) | 0.11 | 0.062 | 0.035 | -945 (-1816; -74) |
| Ratio of IL-10 in *P. aeruginosa*-stimulated and unstimulated maternal blood – change from baseline to visit 2 | -3.48 (12.83) -1.06 (-51.32; 10.23) (-1.93; 0.36) n=17 | -5.07 2.71 (-10.62-0.48) | 0.28 | 2.96 (7.24) 1.03 (-7.55; 19.02) (-0.25; 6.5) n=14 | 3.80 2.90 (-2.16-9.76) | 0.15 | 0.091 | 0.039 | -8.87 (-17.27; -0.47) |
| Analysis performed on the intention-to-treat (ITT) population.  For continuous variables, mean (SD) / median (min; max) / (Q1; Q3) / n are presented. For comparison between arms, Student’s t-test was used for continuous variables. For comparison within arms, paired Student´s t-test was used.  † Interleukin-10 ‡ Adjusting for maternal body mass index (BMI) using Analysis of Covariance (ANCOVA). | | | | | | | | | |
