# Supplementary material for: Oral Lacticaseibacillus rhamnosus GG Exposure During Pregnancy and Effects on Maternal Inflammatory Response—A Blinded, Pilot Randomized, Placebo‐Controlled Study
Source: Am J Reprod Immunol. 2025 Dec 10;94(6):e70190. doi: 10.1111/aji.70190 (PMC12692997; doi:10.1111/aji.70190)
Supplement: Supplementary file 12 — Supplemental Table 6a: IL‐12† levels in maternal blood (ITT ‡ , n = 105) – showing that IL12 levels in both unstimulated maternal blood and E. coli LPS stimulated maternal blood decrease from baseline to visit 2 in the intervention arm and increase in the placebo arm. On the contrary, IL‐12 levels in P. aeruginosa‐stimulated maternal blood increased from visit 2 to 3 in the intervention arm and increased in the placebo arm. [file AJI-94-e70190-s004.docx]

**Supplemental Table 6a. IL-12**^†^ **levels in maternal blood (ITT***^‡^***, n=105) – showing that IL12 levels in both unstimulated maternal blood and *E. coli* LPS stimulated maternal blood decrease from baseline to visit 2 in the intervention arm and increase in the placebo arm. On the contrary, IL-12 levels in *P. aeruginosa-*stimulated maternal blood increased from visit 2 to 3 in the intervention arm and increased in the placebo arm.**

|  | **Intervention (n=53)** | | **Placebo (n=52)** | |  | |
| --- | --- | --- | --- | --- | --- | --- |
| **Variable** | **Mean (SD) Median (Min; Max) (Q1; Q3)** | **p-value within arm** | **Mean (SD) Median (Min; Max) (Q1; Q3)** | **p-value within arm** | **p-value between arms** | **Difference between arms Mean (95% CI)** |
| **IL-12 in unstimulated maternal blood at baseline**  **(number of cytokine-positive monocytes/ml)** | 242.7 (205.2) 170 (0; 1000) (99; 330) n=51 |  | 195.2 (174.4) 170 (0; 1100) (82; 240) n=51 |  | 0.21 | 47.5 (-25.4; 121.3) |
| **IL-12 in unstimulated maternal blood at visit 2**  **(number of cytokine-positive monocytes/ml)** | 169.5 (142.2) 140 (15; 730) (78.5; 220) n=52 |  | 276.3 (261.2) 190 (17; 1300) (110; 380) n=51 |  | 0.0084 | -106.9 (-187.5; -26.2) |
| **IL-12 in unstimulated maternal blood at visit 3**  **(number of cytokine-positive monocytes/ml)** | 276.0 (325.4) 180 (17; 1700) (99; 290) n=47 |  | 315.5 (466.5) 170 (0; 2500) (86; 390) n=46 |  | 0.65 | -39.5 (-205.4; 123.2) |
| **IL-12 in unstimulated maternal blood – change from baseline to visit 2**  **(number of cytokine-positive monocytes/ml)** | -85.7 (192.0) -60 (-540; 270) (-150; 13) n=50 | 0.0025 | 77.6 (299.9) 49 (-780; 1236) (-110; 201) n=50 | 0.072 | 0.0009 | -163.3 (-263.7; -64.4) |
| **IL-12 in *Escherichia coli* LPS*^§^*-stimulated maternal blood at baseline**  **(number of cytokine-positive monocytes/ml)** | 1398 (827) 1300 (85; 3200) (770; 1900) n=51 |  | 1139 (763) 940 (62; 3300) (530; 1500) n=51 |  | 0.10 | 258.7 (-51.5; 570.1) |
| **IL-12 in *E. coli* LPS-stimulated maternal blood at visit 2**  **(number of cytokine-positive monocytes/ml)** | 1140 (843) 960 (130; 4200) (565; 1400) n=52 |  | 1619 (2342) 1300 (180; 17000) (560; 1900) n=51 |  | 0.15 | -479.2 (-1081.7; 103.8) |
| **IL-12 in *E. coli* LPS-stimulated maternal blood at visit 3**  **(number of cytokine-positive monocytes/ml)** | 1954 (1529) 1600 (86; 7200) (930; 2400) n=47 |  | 1748 (1197) 1600 (58; 5900) (960; 2200) n=46 |  | 0.48 | 206.1 (-368.2; 765.1) |
| **IL-12 in *E. coli* LPS-stimulated maternal blood – change from baseline to visit 2**  **(number of cytokine-positive monocytes/ml)** | -236.3 (1077.0) -350 (-2580; 3550) (-1070; 390) n=50 | 0.13 | 481.8 (2457.8) 344 (-2400; 15500) (-530; 900) n=50 | 0.15 | 0.038 | -718 (-1421; -32) |
| **IL-12 in *Lactobacillus paracasei*-stimulated maternal blood at baseline**  **(number of cytokine-positive monocytes/ml)** | 1898 (2525) 1200 (77; 13000) (480; 2100) n=51 |  | 1367 (1236) 1100 (130; 6200) (510; 1800) n=51 |  | 0.19 | 532 (-231; 1308) |
| **IL-12 in *L. paracasei*-stimulated maternal blood at visit 2**  **(number of cytokine-positive monocytes/ml)** | 1285 (1275) 955 (190; 7500) (510; 1500) n=52 |  | 1736 (2086) 1000 (120; 13000) (610; 2100) n=51 |  | 0.19 | -451.1 (-1105.0; 200.4) |
| **IL-12 in *L. paracasei*-stimulated maternal blood at visit 3**  **(number of cytokine-positive monocytes/ml)** | 1567 (1285) 1100 (153; 5600) (640; 2200) n=47 |  | 2048 (2648) 1250 (62; 16000) (720; 2200) n=46 |  | 0.29 | -481.0 (-1305.8; 333.5) |
| **IL-12 in *L. paracasei*-stimulated maternal blood – change from baseline to visit 2**  **(number of cytokine-positive monocytes/ml)** | -616 (2330) -170 (-8800; 5200) (-900; 400) n=50 | 0.067 | 377.0 (2313.1) 80 (-4330; 11500) (-790; 930) n=50 | 0.27 | 0.035 | -993 (-1901; -66) |
| **IL-12 in *L. paracasei*-stimulated maternal blood – change from baseline to visit 3**  **(number of cytokine-positive monocytes/ml)** | -409.2 (2496.1) 100 (-8800; 4690) (-700; 610) n=45 | 0.28 | 670 (2706) 130 (-3200; 14000) (-330; 700) n=45 | 0.095 | 0.047 | -1079 (-2161; -9) |
| **IL-12 *Pseudomonas aeruginosa-*stimulated maternal blood at baseline**  **(number of cytokine-positive monocytes/ml)** | 1304 (986) 1100 (97; 4000) (650; 1600) n=51 |  | 1569 (1714) 1050 (68; 7700) (560; 1600) n=50 |  | 0.35 | -265.3 (-810.0; 287.8) |
| **IL-12 *P. aeruginosa-*stimulated maternal blood at visit 2**  **(number of cytokine-positive monocytes/ml)** | 1162 (1504) 780 (60; 8900) (450; 1300) n=51 |  | 2293 (3035) 1200 (140; 15000) (737; 2400) n=51 |  | 0.014 | -1131 (-2066; -210) |
| **IL-12 *P. aeruginosa-*stimulated maternal blood at visit 3**  **(number of cytokine-positive monocytes/ml)** | 2068 (2207) 1300 (210; 10000) (990; 1900) n=47 |  | 1922 (1903) 1400 (290; 11000) (820; 2500) n=46 |  | 0.74 | 145.7 (-701.2; 993.0) |
| **IL-12 *P. aeruginosa-*stimulated maternal blood – change from visit 2 to visit 3**  **(number of cytokine-positive monocytes/ml)** | 860 (1947) 515 (-5500; 8100) (60; 1150) n=46 | 0.0014 | -192.4 (2943.2) 120 (-10800; 10260) (-750; 860) n=45 | 0.66 | 0.044 | 1053 (27; 2077) |
| **IL-12 *P. aeruginosa-*stimulated maternal blood – change from visit 2 to visit 3**  **(number of cytokine-positive monocytes/ml)** | 754 (1914) 352 (-5490; 8050) (30; 1106) n=46 | 0.0049 | -257.4 (2779.5) 160 (-9650; 9953) (-690; 737) n=45 | 0.53 | 0.043 | 1011 (32; 1986) |
| Analysis performed on the intention-to-treat (ITT) population.  For continuous variables, mean (SD) / median (min; max) / (Q1; Q3) / n are presented. For comparison between arms, Fisher’s non-parametric permutation test was used for continuous variables. For comparison within arms, Fisher´s non-parametric permutation test for matched pairs was used. The confidence interval for the mean difference between arms is based on Fisher’s non-parametric permutation test.  † Interleukin-12  ‡ Intention-to-treat  ***^§^*** Lipopolysaccharide from *Escherichia coli* | | | | | | |

**Supplemental Table 6b. IL-12**^†^ **levels in maternal blood in the previous preterm delivery subgroup (n=37) – Maternal IL-12 levels were lower at visit 2 in the intervention arm than in the placebo arm, both in unstimulated and *L. paracasei*-stimulated blood. Furthermore, the intervention arm showed a greater decrease in IL-12 levels from visit 1 to 2 than the placebo arm, in *L. paracasei*-stimulated blood.**

|  | **Intervention (n=18)** | | | **Placebo (n=19)** | | |  | | |
| --- | --- | --- | --- | --- | --- | --- | --- | --- | --- |
| **Variable** | **Mean (SD) Median (Min; Max) (Q1; Q3)** | **Adjusted means**† **SEM (95% CI)** | **p-value within arm** | **Mean (SD) Median (Min; Max) (Q1; Q3)** | **Adjusted means^‡^ SEM (95% CI)** | **p-value within arm** | **p-value between arms** | **Adjusted p-value^‡^** | **Difference between arms Adjusted means (95% CI)** |
| **IL-12 in unstimulated maternal blood at baseline**  **(number of cytokine-positive monocytes/ml)** | 179.4 (139.4) 125 (0; 440) (89; 310) n=18 | 165.5 37.4 (89.4-241.6) |  | 194.2 (157.3) 150 (19; 600) (82; 260) n=18 | 195.1 36.2 (121.3-268.9) |  | 0.77 | 0.59 | -29.6 (-139.7; 80.5) |
| **IL-12 in unstimulated maternal blood at visit 2**  **(number of cytokine-positive monocytes/ml)** | 146.0 (98.1) 140 (17; 360) (66; 180) n=18 | 133.9 53.3 (25.4-242.4) |  | 319.1 (274.9) 240 (18; 1200) (130; 450) n=19 | 334.1 50.2 (231.9-436.2) |  | 0.017 | 0.013 | -200.1 (-354.9; -45.4) |
| **IL-12 in unstimulated maternal blood at visit 3**  **(number of cytokine-positive monocytes/ml)** | 161.4 (77.4) 165 (17; 280) (120; 210) n=14 | 161.1 93.7 (-32.0-354.1) |  | 314.2 (428.7) 170 (48; 1800) (130; 400) n=15 | 317.3 86.8 (138.5-496.1) |  | 0.20 | 0.25 | -156.2 (-428.1; 115.6) |
| **IL-12 in *L. paracasei-*stimulated maternal blood at baseline**  **(number of cytokine-positive monocytes/ml)** | 1460 (2475) 655 (77; 11000) (420; 1700) n=18 | 987 177 (626-1348) |  | 1037 (747) 840 (180; 3200) (460; 1300) n=18 | 954 172 (604-1304) |  | 0.50 | 0.90 | 32.6 (-489.3; 554.5) |
| **IL-12 in *L. paracasei-*stimulated maternal blood at visit 2**  **(number of cytokine-positive monocytes/ml)** | 928 (754) 665 (210; 2800) (330; 1200) n=18 | 769 245 (271-1268) |  | 1344 (1162) 900 (120; 4500) (540; 1900) n=19 | 1420 231 (950-1889) |  | 0.21 | 0.072 | -650 (-1361; 61) |
| **IL-12 in *L. paracasei-*stimulated maternal blood at visit 3**  **(number of cytokine-positive monocytes/ml)** | 1334 (924) 1050 (420; 3700) (660; 1700) n=14 | 1031 883 (-787-2849) |  | 2290 (4087) 850 (62; 16000) (370; 2200) n=15 | 2495 818 (810-4179) |  | 0.39 | 0.25 | -1463 (-4025; 1098) |
| **IL-12 in *L. paracasei-*stimulated maternal blood – change from baseline to visit 2**  **(number of cytokine-positive monocytes/ml)** | -532 (2206) -15 (-8800; 1720) (-870; 433) n=18 | -217.8 246.5 (-720.0-284.4) | 0.32 | 370.0 (1234.7) 405 (-1900; 2970) (-340; 900) n=18 | 533 239 (46-1020) | 0.22 | 0.14 | 0.043 | -751 (-1477; -25) |
| Analysis performed on the intention-to-treat (ITT) population.  For continuous variables, mean (SD) / median (min; max) / (Q1; Q3) / n are presented. For comparison between arms, Student’s t-test was used for continuous variables. For comparison within arms, paired Student’s t-test was used.  † Interleukin-12 ‡ Adjusting for maternal body mass index (BMI) using Analysis of Covariance (ANCOVA). | | | | | | | | | |

**Supplemental Table 6c. IL-12**^†^ **levels in maternal blood in the control subgroup (n=33) – the ratio of IL-12 levels in *Pseudomonas aeruginosa*-stimulated and unstimulated maternal blood increases from visit 2 to 3 in the intervention arm and decreases in the placebo arm.**

|  | **Intervention (n=17)** | | | **Placebo (n=16)** | | |  | | |
| --- | --- | --- | --- | --- | --- | --- | --- | --- | --- |
| **Variable** | **Mean (SD) Median (Min; Max) (Q1; Q3)** | **Adjusted means**† **SEM (95% CI)** | **p-value within arm** | **Mean (SD) Median (Min; Max) (Q1; Q3)** | **Adjusted means^‡^ SEM (95% CI)** | **p-value within arm** | **p-value between arms** | **Adjusted p-value^‡^** | **Difference between arms Adjusted means (95% CI)** |
| **IL-12 in unstimulated maternal blood at baseline**  **(number of cytokine-positive monocytes/ml)** | 264.8 (209.4) 175 (32; 670) (98; 405) n=16 | 268.9 41.9 (183.1-354.7) |  | 174.8 (82.3) 190 (0; 300) (112.5; 235) n=16 | 170.7 41.9 (84.9-256.5) |  | 0.13 | 0.12 | 98.2 (-27.6; 223.9) |
| **IL-12 in unstimulated maternal blood at visit 2**  **(number of cytokine-positive monocytes/ml)** | 160.5 (88.6) 110 (64; 380) (92; 220) n=17 | 160.5 60.7 (36.4-284.5) |  | 288.1 (329.9) 170 (21; 1300) (110; 320) n=16 | 288.2 62.7 (160.1-416.3) |  | 0.15 | 0.17 | -127.7 (-312.0; 56.6) |
| **IL-12 in unstimulated maternal blood at visit 3**  **(number of cytokine-positive monocytes/ml)** | 255.1 (253.5) 145 (39; 810) (62.5; 350) n=16 | 249.8 135.3 (-27.8-527.4) |  | 444.1 (698.6) 165 (0; 2500) (86; 390) n=14 | 450.2 145.4 (151.7-748.6) |  | 0.35 | 0.34 | -200.4 (-624.2; 223.5) |
| **IL-12 in *P. aeruginosa-*stimulated maternal blood at baseline**  **(number of cytokine-positive monocytes/ml)** | 1742 (1196) 1550 (160; 4000) (970; 2550) n=16 | 1867 517 (809-2925) |  | 2522 (2538) 1450 (68; 7700) (870; 3750) n=16 | 2397 517 (1339-3454) |  | 0.28 | 0.49 | -530 (-2080; 1021) |
| **IL-12 in *P. aeruginosa-*stimulated maternal blood at visit 2**  **(number of cytokine-positive monocytes/ml)** | 1811 (2426) 880 (110; 8900) (350; 1900) n=17 | 1854 783 (255-3453) |  | 3489 (3637) 1350 (550; 12000) (935; 6100) n=16 | 3443 809 (1791-5094) |  | 0.13 | 0.18 | -1589 (-3964; 786) |
| **IL-12 in *P. aeruginosa-*stimulated maternal blood at visit 3**  **(number of cytokine-positive monocytes/ml)** | 2895 (3262) 1300 (310; 10000) (1100; 3650) n=16 | 2950 696 (1522-4378) |  | 2500 (1622) 2150 (450; 5200) (1200; 3700) n=14 | 2437 748 (903-3972) |  | 0.67 | 0.63 | 512 (-1667; 2692) |
| **Ratio of IL-12 in *P. aeruginosa-*stimulated and unstimulated maternal blood – change from visit 2 to visit 3** | 7.17 (17.80) 2.65 (-23.24; 50.32) (-4.49; 17.5) n=16 | 7.41 4.39 (-1.62-16.44) | 0.13 | -8.22 (15.23) -1.23 (-49.17; 6.25) (-13.18; 1.22) n=13 | -8.52 4.91 (-18.61-1.57) | 0.076 | 0.020 | 0.027 | 15.9 (1.9; 29.9) |
| Analysis performed on the intention-to-treat (ITT) population.  For continuous variables, mean (SD) / median (min; max) / (Q1; Q3) / n are presented. For comparison between arms, Student’s t-test was used for continuous variables. For comparison within arms, paired Student´s t-test was used.  † Interleukin-12 ‡ Adjusting for maternal body mass index (BMI) using Analysis of Covariance (ANCOVA). | | | | | | | | | |
