# Supplementary material for: Oral Lacticaseibacillus rhamnosus GG Exposure During Pregnancy and Effects on Maternal Inflammatory Response—A Blinded, Pilot Randomized, Placebo‐Controlled Study
Source: Am J Reprod Immunol. 2025 Dec 10;94(6):e70190. doi: 10.1111/aji.70190 (PMC12692997; doi:10.1111/aji.70190)
Supplement: Supplementary file 13 — Supplemental File 1: aji70190‐sup‐0013‐SuppMat.docx [file AJI-94-e70190-s011.docx]

### Blood analysis

#### **Intracellular cytokines in stimulated monocytes**

Cytokine production in monocytes after whole blood stimulation was assessed with flow cytometry by intracellular staining of monocytes. Briefly, heparinized blood was diluted to 1/10 in RPMI1640 cell culture medium (BioWhittaker Inc., Walkersville, MD, USA) with 1% L-glutamine, 1% mercaptoethanol and 0.1 mg/ mL gentamicin. This was mixed with stimulators in the presence of Golgistop (BD). LPS from *Escherichia coli* (final concentration 2 µg/mL) and strains of *Lactobacillus paracasei* and *Pseudomonas aeruginosa* (both at a final concentration of 10^8^/mL) were used as stimulators. After 4 hours of incubation at 37 ^o^C and 5 % CO_2,_ the cells were harvested by centrifugation at 400 x g for five minutes. The red cells in the cell pellet were lysed, using an ammonium chloride buffer (pH 7.3) according to standard procedures, and washed in FACSFlow Sheath Fluid (BD Biosciences). They were subsequently permeabilized and fixed, using Cytofix/Cytoperm according to the manufacturer’s (BD Biosciences) instructions. The cells were stained with fluorophore-conjugated antibodies: fluorescein isothiocyanate (FITC)-conjugated anti-TNF-α, phycoerythrin (PE)-conjugated anti-IL-12, peridinin-chlorophyll-protein (PerCP)-conjugated anti-CD14 and allophycocyanin (APC)-conjugated anti-IL-10 (all from BD Biosciences). After incubation for 15 minutes at 4 ^o^C and washing with buffer B, the cells were resuspended in 200 µL of washing solution A. A defined volume was analyzed by collecting the sample for 120 seconds with the flow cytometer set at the flow rate of each analysis. The results were analyzed using DIVA software and expressed as the number of positive cells per mL.

#### **Flow cytometry for measuring T-, B-, and natural killer cells (TBNK)**

Levels of lymphocyte subpopulations in whole blood were determined by flow cytometry. A 50-µl aliquot of whole blood collected in EDTA tubes was transferred to TruCount tubes and labeled with FITC-CD3/PE-CD16+CD56/PerCP-CD45/PECy7-CD4/APCCD19/APCCy7-CD8, according to manufacturer’s instructions (BD Biosciences). Cells were analyzed with a FACSCanto II flow cytometer (BD Biosciences) that was checked daily with Cytometer Setup and Tracking (CST) beads, using DIVA software. Lymphocyte gates inside scatter versus CD45 plots and quadrant statistics from the six-color analysis were generated with Canto software (BD Biosciences), followed by manual adjustments if needed. The results for each subpopulation were expressed as the percentage and the number (10^9^/L) of lymphocytes.

#### **Lymphocyte subpopulations**

Blood collected in heparin tubes was lysed with ammonium chloride, washed in washing solution A (PBS with 3 % (v/v) fetal bovine serum, 0.09 % (w/v) sodium azide and 0.5 mM EDTA) and blocked with blocking solution A (10 % (v/v), AB serum and 20 % (v/v) unconjugated mouse serum) (BD Biosciences, Mountain View, CA, USA). Before staining with FoxP3, the cells were fixed and permeabilized using FixPerm (AH Diagnostics AB, Solna, Sweden), according to the manufacturer’s instructions. After cold incubation for 30 minutes, the cells were washed with FACSFlow, followed by Perm buffer (BD Biosciences). They were then blocked with normal rat serum (AH Diagnostics AB). The following antibodies were added to the cells: fluorescein isothiocyanate (FITC)-conjugated anti-CD25, PerCP-conjugated anti-CD4, APC-conjugated anti-CD3 (all from BD Biosciences) and either phycoerythrin (PE)-conjugated anti-FoxP3 or rat anti-IgG2a (AH Diagnostics). Cells were incubated at 4-8℃ for 30 minutes, then washed in Perm buffer, after which washing solution A was added.

Cell analysis was carried out on a flow cytometer (FACSCanto II, BD Biosciences, see above). First, a lymphocyte gate was set manually in the forward-scatter versus side-scatter diagram. Dot plots and quadrant statistics from the four-color analysis were then set and analyzed using DIVA software. Finally, the results for each subpopulation were expressed as the percentage and, based on the TBNK assay, as the number (x 10^9^/L) of lymphocytes.
