# Supplementary material for: Oral Lacticaseibacillus rhamnosus GG Exposure During Pregnancy and Effects on Maternal Inflammatory Response—A Blinded, Pilot Randomized, Placebo‐Controlled Study
Source: Am J Reprod Immunol. 2025 Dec 10;94(6):e70190. doi: 10.1111/aji.70190 (PMC12692997; doi:10.1111/aji.70190)
Supplement: Supplementary file 14 — Supplemental File 2: aji70190‐sup‐0014‐SuppMat.docx [file AJI-94-e70190-s014.docx]

**Gating strategies in the ICC study.**

#### **Flow cytometry for measuring T-, B-, and natural killer cells (TBNK)**


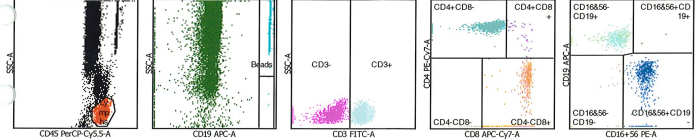


1 2 3 4 5

1 Lymphocytes defined by Side scatter versus CD45.

2 Trucount beads excluded.

3 Definition of T lymphocytes.

4 Definition of CD4 and CD8.

5 Definition of CD19 of CD16+56+.

#### **Lymphocyte subpopulations**


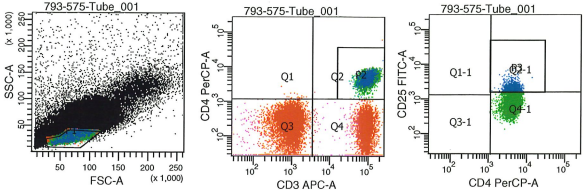


1 2 3


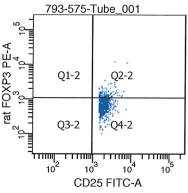


4

1 Lymphocytes defined by Side scatter versus Forward scatter.

2 Definition of CD3+4+.

3 Definition of CD4+25+.

4 Definition of CD25+FOXP3+.

#### **Intracellular cytokines in stimulated monocytes**


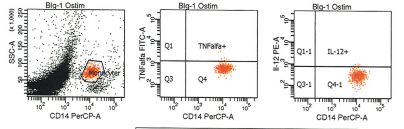


1 2 3


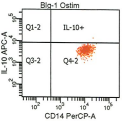

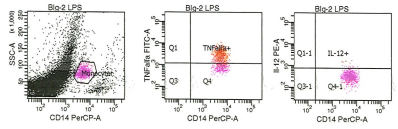


4 5 6 7


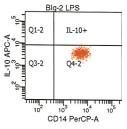


8

1 (and 5) Definition of monocytes by Side scatter and CD14.

2 (and 6) Definition of TNFα+ monocytes.

3 (and 7) Definition of IL12+ monocytes.

4 (and 8) Definition of IL10+ monocytes.
